# Ovarian microenvironment: challenges and opportunities in protecting against chemotherapy-associated ovarian damage

**DOI:** 10.1093/humupd/dmae020

**Published:** 2024-06-28

**Authors:** Yican Guo, Liru Xue, Weicheng Tang, Jiaqiang Xiong, Dan Chen, Yun Dai, Chuqing Wu, Simin Wei, Jun Dai, Meng Wu, Shixuan Wang

**Affiliations:** Department of Obstetrics and Gynecology, Tongji Hospital, Tongji Medical College, Huazhong University of Science and Technology, Wuhan, Hubei, China; National Clinical Research Center for Obstetrical and Gynecological Diseases, Wuhan, Hubei, China; Key Laboratory of Cancer Invasion and Metastasis, Ministry of Education, Wuhan, Hubei, China; Department of Obstetrics and Gynecology, Tongji Hospital, Tongji Medical College, Huazhong University of Science and Technology, Wuhan, Hubei, China; National Clinical Research Center for Obstetrical and Gynecological Diseases, Wuhan, Hubei, China; Key Laboratory of Cancer Invasion and Metastasis, Ministry of Education, Wuhan, Hubei, China; Department of Obstetrics and Gynecology, Tongji Hospital, Tongji Medical College, Huazhong University of Science and Technology, Wuhan, Hubei, China; National Clinical Research Center for Obstetrical and Gynecological Diseases, Wuhan, Hubei, China; Key Laboratory of Cancer Invasion and Metastasis, Ministry of Education, Wuhan, Hubei, China; Department of Obstetrics and Gynecology, Zhongnan Hospital of Wuhan University, Wuhan, China; Department of Obstetrics and Gynecology, Tongji Hospital, Tongji Medical College, Huazhong University of Science and Technology, Wuhan, Hubei, China; National Clinical Research Center for Obstetrical and Gynecological Diseases, Wuhan, Hubei, China; Key Laboratory of Cancer Invasion and Metastasis, Ministry of Education, Wuhan, Hubei, China; Department of Obstetrics and Gynecology, Tongji Hospital, Tongji Medical College, Huazhong University of Science and Technology, Wuhan, Hubei, China; National Clinical Research Center for Obstetrical and Gynecological Diseases, Wuhan, Hubei, China; Key Laboratory of Cancer Invasion and Metastasis, Ministry of Education, Wuhan, Hubei, China; Department of Obstetrics and Gynecology, Tongji Hospital, Tongji Medical College, Huazhong University of Science and Technology, Wuhan, Hubei, China; National Clinical Research Center for Obstetrical and Gynecological Diseases, Wuhan, Hubei, China; Key Laboratory of Cancer Invasion and Metastasis, Ministry of Education, Wuhan, Hubei, China; Department of Obstetrics and Gynecology, Tongji Hospital, Tongji Medical College, Huazhong University of Science and Technology, Wuhan, Hubei, China; National Clinical Research Center for Obstetrical and Gynecological Diseases, Wuhan, Hubei, China; Key Laboratory of Cancer Invasion and Metastasis, Ministry of Education, Wuhan, Hubei, China; Department of Obstetrics and Gynecology, Tongji Hospital, Tongji Medical College, Huazhong University of Science and Technology, Wuhan, Hubei, China; National Clinical Research Center for Obstetrical and Gynecological Diseases, Wuhan, Hubei, China; Key Laboratory of Cancer Invasion and Metastasis, Ministry of Education, Wuhan, Hubei, China; Department of Obstetrics and Gynecology, Tongji Hospital, Tongji Medical College, Huazhong University of Science and Technology, Wuhan, Hubei, China; National Clinical Research Center for Obstetrical and Gynecological Diseases, Wuhan, Hubei, China; Key Laboratory of Cancer Invasion and Metastasis, Ministry of Education, Wuhan, Hubei, China; Department of Obstetrics and Gynecology, Tongji Hospital, Tongji Medical College, Huazhong University of Science and Technology, Wuhan, Hubei, China; National Clinical Research Center for Obstetrical and Gynecological Diseases, Wuhan, Hubei, China; Key Laboratory of Cancer Invasion and Metastasis, Ministry of Education, Wuhan, Hubei, China

**Keywords:** ovarian microenvironment, chemotherapy, fertility preservation, ovarian reserve, protective therapies, extracellular matrix, vascular system, immune, stem cell

## Abstract

**BACKGROUND:**

Chemotherapy-associated ovarian damage (CAOD) is one of the most feared short- and long-term side effects of anticancer treatment in premenopausal women. Accumulating detailed data show that different chemotherapy regimens can lead to disturbance of ovarian hormone levels, reduced or lost fertility, and an increased risk of early menopause. Previous studies have often focused on the direct effects of chemotherapeutic drugs on ovarian follicles, such as direct DNA damage-mediated apoptotic death and primordial follicle burnout. Emerging evidence has revealed an imbalance in the ovarian microenvironment during chemotherapy. The ovarian microenvironment provides nutritional support and transportation of signals that stimulate the growth and development of follicles, ovulation, and corpus luteum formation. The close interaction between the ovarian microenvironment and follicles can determine ovarian function. Therefore, designing novel and precise strategies to manipulate the ovarian microenvironment may be a new strategy to protect ovarian function during chemotherapy.

**OBJECTIVE AND RATIONALE:**

This review details the changes that occur in the ovarian microenvironment during chemotherapy and emphasizes the importance of developing new therapeutics that protect ovarian function by targeting the ovarian microenvironment during chemotherapy.

**SEARCH METHODS:**

A comprehensive review of the literature was performed by searching PubMed up to April 2024. Search terms included ‘ovarian microenvironment’ (ovarian extracellular matrix, ovarian stromal cells, ovarian interstitial, ovarian blood vessels, ovarian lymphatic vessels, ovarian macrophages, ovarian lymphocytes, ovarian immune cytokines, ovarian oxidative stress, ovarian reactive oxygen species, ovarian senescence cells, ovarian senescence-associated secretory phenotypes, ovarian oogonial stem cells, ovarian stem cells), terms related to ovarian function (reproductive health, fertility, infertility, fecundity, ovarian reserve, ovarian function, menopause, decreased ovarian reserve, premature ovarian insufficiency/failure), and terms related to chemotherapy (cyclophosphamide, lfosfamide, chlormethine, chlorambucil, busulfan, melphalan, procarbazine, cisplatin, doxorubicin, carboplatin, taxane, paclitaxel, docetaxel, 5-fluorouraci, vincristine, methotrexate, dactinomycin, bleomycin, mercaptopurine).

**OUTCOMES:**

The ovarian microenvironment shows great changes during chemotherapy, inducing extracellular matrix deposition and stromal fibrosis, angiogenesis disorders, immune microenvironment disturbance, oxidative stress imbalances, ovarian stem cell exhaustion, and cell senescence, thereby lowering the quantity and quality of ovarian follicles. Several methods targeting the ovarian microenvironment have been adopted to prevent and treat CAOD, such as stem cell therapy and the use of free radical scavengers, senolytherapies, immunomodulators, and proangiogenic factors.

**WIDER IMPLICATIONS:**

Ovarian function is determined by its ‘seeds’ (follicles) and ‘soil’ (ovarian microenvironment). The ovarian microenvironment has been reported to play a vital role in CAOD and targeting the ovarian microenvironment may present potential therapeutic approaches for CAOD. However, the relation between the ovarian microenvironment, its regulatory networks, and CAOD needs to be further studied. A better understanding of these issues could be helpful in explaining the pathogenesis of CAOD and creating innovative strategies for counteracting the effects exerted on ovarian function. Our aim is that this narrative review of CAOD will stimulate more research in this important field.

**REGISTRATION NUMBER:**

Not applicable.

## Graphical abstract

**Figure:**
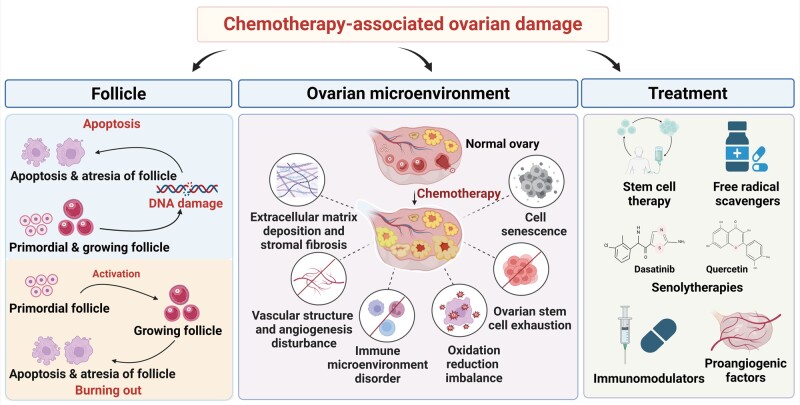
**Chemotherapy causes an imbalance of the ovarian microenvironment leading to chemotherapy-associated ovarian damage and dysfunction, and further research is needed to explore the possible protective treatments.** Created with BioRender.com, with permission.

## Introduction

In 2024, over 2 million new cancer cases are projected to occur in the USA, and the 5-year relative survival rate for all cancers combined has increased from 49% for diagnoses during the mid-1970s to 69% during 2013–2019 (Siegel *et al.*, 2024). Compared to the general population, women are 38% less likely to become pregnant after a cancer diagnosis and its treatment (Anderson *et al.*, 2018). The ovary is responsible for fertility and for maintaining the woman’s endocrinological balance until menopause. Chemotherapy can lead to ovarian hormone level disturbance, persistent abnormal menses, amenorrhea, infertility, and an increased risk of early menopause (van Dorp *et al.*, 2018). The impacts of oestrogen deficiency, such as menopausal symptoms, osteoporosis, cardiovascular disease, and cognitive decline, are also a critical aspect of the longer-term side of chemotherapy-associated ovarian damage (CAOD) (Lobo, 2017). Therefore, understanding the biological mechanisms of CAOD and developing new ovarian preservation strategies are paramount.

The proposed mechanisms underlying CAOD primarily involve the induction of DNA cross-link formation within oocytes or granulosa cells, triggering apoptosis, or the overactivation and subsequent exhaustion of dormant primordial follicles, ultimately leading to a decreased number of follicles (Soleimani *et al.*, 2011; Kalich-Philosoph *et al.*, 2013; Li *et al.*, 2014; Chang *et al.*, 2015; Luan *et al.*, 2019; Nguyen *et al.*, 2019). Ovarian function is determined by its ‘seeds’ (follicles) and ‘soil’ (ovarian microenvironment). The ovarian microenvironment provides nutritional support and signal transportation for the growth and development of follicles, ovulation, and corpus luteum formation, and the close interaction between follicles and ovarian microenvironment determines the fate of follicles and thus the ovarian lifespan (Ahmed *et al.*, 2020). The ovarian microenvironment comprises: the extracellular matrix (ECM); the ovarian stromal cells and cytokines; the vascular system, which consists of blood vessels and lymphatics; the immune system, including immune cells, chemokines, and inflammatory cytokines; ovarian stem cells; and others, including metabolic products (e.g. amino acid metabolites, glucose metabolites, trance elements), nerves, hormones, etc. Emerging evidence has highlighted the crucial role of the ovarian microenvironment in CAOD. Some studies show that chemotherapy depletes follicles through inducing ovarian stromal fibrosis and destroying ovarian vascular structure and function (Meirow *et al.*, 2007; Oktem and Oktay, 2007; Bar-Joseph *et al.*, 2011; Pascuali *et al.*, 2018). Chemotherapy also leads to an imbalance of immune and oxidative stress in the ovarian microenvironment, further inducing follicle apoptosis and loss (Deng *et al.*, 2021; Dinc *et al.*, 2023). Chemotherapy may induce the apoptosis of ovarian stem cells that might be able to renew oocytes and remodel ovarian function (Jiang *et al.*, 2019; Wu *et al.*, 2019). After chemotherapy, senescent cells accumulate in the ovarian microenvironment and secrete many proteins related to the senescence-associated secretion phenotype (SASP), which contributes to ovarian ageing (Hense *et al.*, 2022). Hence, there is now a pressing need to explore the role of the microenvironment in CAOD and develop methods to improve the balance of the ovarian microenvironment during chemotherapy.

This review introduces the physiological role of the ovarian microenvironment, details its changes during chemotherapy and the mechanisms of damage to ovarian function, and summarizes proposed protective treatments targeting the ovarian microenvironment.

## Methods

A comprehension literature review was carried out to identify relevant articles pertaining to changes in the ovarian microenvironment induced by chemotherapy and to new approaches of its protection. All authors contributed to the search and to establish the inclusion and exclusion criteria. As this was an analysis of published data, approval of an ethics committee is not relevant.

The research was performed using PubMed Central as sources, and identified peer-reviewed English publications for human and animal up to April 2024. Searches were performed by adopting the three groups of main terms. The first group included ‘ovarian microenvironment’ (ovarian ECM, ovarian stromal cells, ovarian interstitial, ovarian cytokines, ovarian blood vessels, ovarian lymphatic vessels, ovarian macrophages, ovarian lymphocytes, ovarian immune cytokines, ovarian oxidative stress, ovarian reactive oxygen species (ROS), ovarian senescence cells, ovarian SASPs, ovarian oogonial stem cells, ovarian germline stem cells, ovarian stem cells), the second group included ovarian function-related terms (reproductive health, fertility, infertility, fecundity, ovarian damage, ovarian reserve, ovarian function, decreased ovarian reserve (DOR), premature ovarian failure (POF), premature ovarian insufficiency (POI), menopause), and the third group was chemotherapy-related terms (cyclophosphamide (CTX), lfosfamide, chlormethine, chlorambucil, busulfan (BUL), melphalan, procarbazine, cisplatin (CIS), carboplatin, taxane, paclitaxel, docetaxel, doxorubicin (DOX), 5-fluorouraci, vincristine, methotrexate, dactinomycin, bleomycin, mercaptopurine). In certain areas where human research was limited, data from animal studies were used.

All relevant articles were carefully evaluated. Initially, titles and abstracts were assessed to evaluate the eligibility of the studies. After this selection, the authors proceeded with the complete reading of the papers to identify those relevant for final inclusion. Reference lists of these papers were checked to identify other studies that should be included in this review. Manuscripts were selected concerning changes in the ovarian microenvironment in response to various chemotherapeutic regimens in the context of reproduction, while those concerning chemotherapeutic drugs that only induced changes of the follicle itself were excluded from the review. Manuscripts describing new approaches of CAOD protection, which improved the ovarian microenvironment, were included. To focus the scope of the current review, case reports, duplicate articles, opinion papers, editorials, and congress abstracts were excluded.

## Ovarian microenvironment

**Figure 1.**
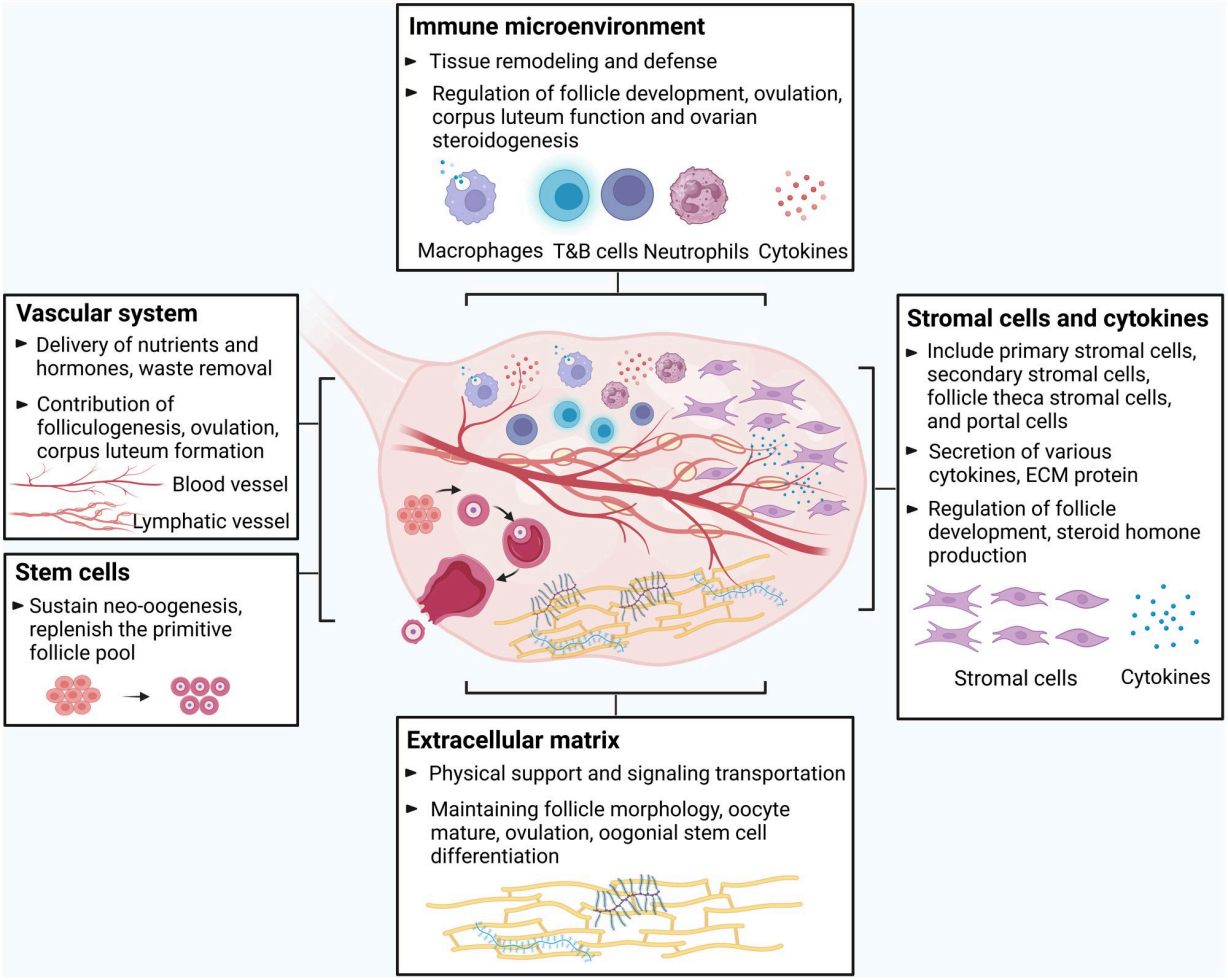
**Schematic of the various contributors to the ovarian microenvironment.** ECM, extracellular matrix. Created with BioRender.com, with permission.

Historically, research on ovarian function has mainly focused on follicles, revealing that the essence of ovarian dysfunction is a decrease in the number and quality of follicles, but recently the ovarian microenvironment has become an exciting new frontier for research as it seems to hold critical keys to understand the complexity of ovarian function. In this review, we mainly discuss the ECM, ovarian stromal cells and cytokines, the vascular system, immune components, and ovarian stem cells, all of which are essential for follicle development and functional maintenance (Fig. 1).

### Ovarian extracellular matrix

The ovarian ECM provides physical support for follicle development and induces intracellular biochemical signaling pathways to maintain or modify the morphology, differentiation, homeostasis, and mechanical properties of the ovarian tissue (Monslow *et al.*, 2015). Recent proteomic studies have revealed that the ECM of human ovarian cortex comprises 46 core matrisome proteins (collagens, glycoproteins, and proteoglycans) and 39 matrisome-associated proteins (ECM-affiliated proteins, ECM regulators, and secreted factors) known to regulate and remodel the ECM (Ouni *et al.*, 2019). Collagen is the main components of the ovarian ECM and represents 49% of the total matrisome proteins (Ouni *et al.*, 2019). Collagen deposition is mainly observed at the outer edge of human ovary, decreasing towards the inner side (Grosbois *et al.*, 2023). Elastin is primarily found at the cortex-medulla border, particularly near blood vessels, and to a lesser extent within human cortical stroma. Fibronectin and laminin are broadly expressed throughout the human stromal compartment.

Examination of the spatiotemporal evolution of collagen deposition in human ovary under brightfield and polarized light microscopy revealed that collagen level increased with age, while fibrillin-1 and emilin-1 declined (Ouni *et al.*, 2020). Collagen and elastin peak in reproductive-age women compared to prepubertal and menopausal individuals. In another study, micro-scale analysis revealed that ovarian ECM underwent distinct changes across different life stages (Ouni *et al.*, 2021). Prepuberty is marked by thin fibers assembled into thin bundles, while reproductive age sees a densification into thickest bundles. Menopause exhibits a tighter network organization, suggesting age-related ECM changes.

Growing evidence suggests that the ovarian ECM regulates follicle formation, growth, and ovulation. Specifically, the ovarian cortex furnishes a stiff matrix conducive to primate primordial follicle growth and survival (Hornick *et al.*, 2012). Within mouse primordial follicles, oocytes experience compression from surrounding granulosa cells, which secret ECM proteins, resulting in elevated mechanical pressure essential for maintaining follicle dormancy (Nagamatsu *et al.*, 2019). Experimentally, loosening of the ovarian ECM via collagenase treatment induces mouse follicle activation, concurrent with nuclear export of FOXO3 and activation of the phosphoinositide 3-kinase (PI3K)/serine-threonine kinase (AKT) pathway (Nagamatsu *et al.*, 2019). In addition, both the Hippo signaling pathway and the PI3K/AKT signaling pathway are involved in the activation of human and mouse primordial follicles induced by mechanical force on ovarian ECM (Kawamura *et al.*, 2013; Grosbois and Demeestere, 2018; Devos *et al.*, 2020). As human follicles grow, they move to the less dense ovarian medulla because this is most permissive to follicular expansion and maturation (Ouni *et al.*, 2020). Human secondary follicles reorient the majority of collagen fibers to below 50° to induce directional fiber remodeling and folliculogenesis compared to follicles at earlier stages of development (Ouni *et al.*, 2021). As they reach the preovulatory stage, the LH surge stimulates human antral follicles to vigorously remodel the ECM through proteolytic degradation (via matrix metalloproteinases (MMPs)), which degrades the apical follicular connective tissue and facilitates follicular rupture (Fiorentino *et al.*, 2023). The development and luteolysis of the corpus luteum depend upon the precise remodeling of the ECM. When human follicular tissue remodels into the corpus luteum, basement membrane (type IV) collagen is replaced by fibrillar (type I) collagen (Li *et al.*, 2020). Type I collagen (COL1A1 and COL1A2) is more abundant in bovine regressing corpus luteum than in the functional corpus luteum, reinforcing the importance of type I collagen in luteal development (Favero *et al.*, 2019). ECM is also involved in the regulation of oogonial stem cell differentiation and oocyte formation. Specifically, the Arg-Gly-Asp motif-binding integrin subunits on the surface of mouse oogonial stem cells interact with type I and type IV collagen in the ovarian stroma to upregulate the levels of meiosis and oocyte formation (MacDonald *et al.*, 2019). In contrast, human oogonial stem cells are unresponsive to a collagen-based ECM but produce significantly more *in vitro*-derived oocytes when cultured on laminin (MacDonald *et al.*, 2019). These data indicate that the ovarian ECM acts in a species-specific manner to control oogonial stem cell differentiation in adult mouse and human ovaries.

Mechanical properties of the ovarian ECM play a crucial role in supporting follicle survival and folliculogenesis, and any alterations to its mechanical properties could contribute to ovarian disorders, such as PCOS and POI. Ovaries from patients with PCOS increase collagen deposition, and have a thickened cortex and altered ECM composition that probably creates a biomechanically non-permissive environment for follicle recruitment and growth (Hughesdon, 1982; Takahashi *et al.*, 1994; Papachroni *et al.*, 2010). Wood *et al.* (2015) used multi-modal magnetic resonance elastography to reveal that patients with a diagnosis of PCOS had stiffer ovaries than those of age-matched controls (Wood *et al.*, 2015). Ovarian laparoscopic drilling, a clinical treatment for PCOS, may destruct the thickened cortical and subcortical stroma, thereby inducing ovulation and promoting follicle growth in patients with PCOS (Seow *et al*., 2020; Abu Hashim, 2015). Moreover, patients with POI display a highly variable ovarian cortical stiffness and a diminished follicle pool (Li *et al.*, 2010; Mendez *et al.*, 2022). Recent clinical studies have shown that *in vitro* ovarian fragmentation with AKT stimulators could activate primordial follicles, and patients with POI deliver healthy babies following IVF after auto-transplanted ovarian fragmentation (Kawamura *et al.*, 2013; Suzuki *et al.*, 2015; Zhai *et al.*, 2016). Moreover, utilizing *in vitro* ovarian cortical fragmentation alone, followed by immediate auto-transplantation, is sufficient to disrupt the Hippo signaling and promote follicle growth in patients with POI and DOR (Lunding *et al.*, 2019; Kawamura *et al.*, 2020; Tanaka *et al.*, 2020; Mendez *et al.*, 2022).

### Ovarian stromal cells and cytokines

The majority of the ovarian stroma is composed of a mixed population of incompletely characterized fibroblasts, commonly referred to as stromal cells, which are grouped into four main types, namely, primary stromal cells, secondary stromal cells, follicle theca stromal cells, and portal cells (Reeves, 1971). Recent human single-cell RNA-sequencing studies have confirmed the presence of multiple ovarian stromal cell clusters, but a comprehensive characterization of stromal cell types is lacking (Fan *et al.*, 2019; Wagner *et al.*, 2020; Wu *et al.*, 2024). The distribution and subtypes of ovarian stromal cells may be affected by cyclic structural changes during follicle growth, ovulation, and luteal development. During follicle development, rodent ovarian stromal cells multiply and differentiate into inner theca cells and outer myofibroblast, the former maintaining integrity of the follicle structure while the latter secretes ECM to participate in the formation of follicular capillaries (Cui *et al.*, 2020; Secchi *et al.*, 2021). Ovarian stromal/theca cells provide androgen to support mammalian pre-antral follicular growth and regulate the proliferation and apoptosis of granulosa cells (Young and McNeilly, 2010; Qiu *et al.*, 2014). Additionally, ovarian stromal cell-feeding layers have been demonstrated to have a positive effect on the *in vitro* survival and/or growth of preantral follicles in both mice and women (Dath *et al.*, 2011; Tingen *et al.*, 2011; Grubliauskaite *et al.*, 2024). Integration of ovarian stromal cells from human fresh medullary tissue into the artificial ovary resulted in higher viability and improved graft vascularization (Soares *et al.*, 2015).

Ovarian stromal cells have the capacity to produce cytokines that actively participate in pivotal ovarian physiological processes (Sirotkin, 2011). Stromal cell-derived factor-1 (SDF-1/CXCL12) and its receptor CXC motif receptor 4 (CXCR4) are expressed in ovarian stroma and follicles (Adamczak *et al.*, 2021). Interactions between SDF-1 and CXCR4 have been suggested to play an essential role in homing primordial germ cells (PGCs) to genital ridges (Doitsidou *et al.*, 2002), with SDF-1 mutant mice displaying delayed migration and decreased numbers of PGCs in the gonads (Ara *et al.*, 2003). A subsequent study suggested the SDF-1/CXCR4 signaling pathway exerted a significant influence on preserving the size and longevity of the mouse primordial follicle pool (Holt *et al.*, 2006). Besides, in human follicular fluid (FF) during IVF, the rates of oocyte recovery increased with higher concentrations of SDF-1 (Nishigaki *et al.*, 2011). A similar result was established in equine, bovine, sheep, and swine (Sayasith and Sirois, 2014; Zhang *et al.*, 2018; Basini *et al.*, 2020). These results suggest that SDF-1 exerts important positive influences on the ovulatory process and follicle development. Additionally, a study involving single-nucleotide polymorphisms (SNPs) conducted on 111 Chinese patients with POF and 183 healthy controls revealed that the polymorphism rs1801157 in CXCL12 exhibited a suggestive association with POF (Wang *et al.*, 2011). SDF-1/CXCR4 signaling has been implicated in the pathogenesis of PCOS by inhibiting ovarian granulosa cell apoptosis in rats (Jin *et al.*, 2021).

Bone morphogenetic protein 4 (BMP-4) and BMP-7, derived from ovarian stromal cells, play essential roles in promoting follicle growth and enhancing follicle survival (Shimizu *et al.*, 2012). These proteins are crucial for the specification, migration, and maintenance of PGCs in mice and humans (Saitou *et al.*, 2002; Abir *et al.*, 2008). In mice with a null mutation for BMP-4 or BMP-7, the number of PGCs was significantly reduced (Lawson *et al.*, 1999; Ross *et al.*, 2007). Additionally, BMP-4 induces the differentiation of mouse ovarian stem cells into oocytes via small mothers against decapentaplegic1 (SMAD1)/SMAD5/SMAD8 signaling (Park *et al.*, 2013). In addition, BMP-4 and BMP-7 have been implicated as regulators of the transition of primordial follicles to primary follicles, and in follicle growth in various species including sheep (Foroughinia *et al.*, 2017), rats (Lee *et al.*, 2001), mice (Tanwar *et al.*, 2008), rabbits (Xie *et al.*, 2014), bovines (da Cunha *et al.*, 2018), and human (Abir *et al.*, 2008). BMP-4 and BMP-7 also act as regulators in cumulus–oocyte complex (COC) expansion and communication between human granulosa cells (Zhang *et al.*, 2016). They also modulate the production of estradiol and progesterone induced by FSH or insulin-like growth factor (IGF) in sheep and mouse granulosa cells (Foroughinia *et al.*, 2017; Liu *et al.*, 2017). In addition, BMP-4 and BMP-7 suppress granulosa cell apoptosis by inhibiting the release of caspase-activated DNase or alleviating endoplasmic reticulum stress in bovine and chicken (Kayamori *et al.*, 2009; Yao *et al.*, 2020).

Ovarian stromal cells are known to express leukemia inhibitory factor (LIF), which plays pivotal roles in growth of the mammal primordial follicle, ovulation, steroidogenesis, and early embryo development (Cadoret *et al.*, 2021; Pena *et al.*, 2023). Studies indicated that LIF concentrations in FF were decreased in patients with PCOS, and that LIF levels could act as a biomarker for predicting outcomes of IVF with embryo transfer (Ledee-Bataille *et al.*, 2001; Li *et al.*, 2018). Furthermore, the frequency of the LIF gene mutation in infertile women is significantly higher compared to fertile controls (Novotny *et al.*, 2009; Vagnini *et al.*, 2019).

The level of monocyte chemotactic protein-1 (MCP-1) in human ovarian stroma increases from the preovulatory to the late ovulatory phase and declines during the postovulatory phase (Dahm-Kahler *et al.*, 2009). High concentrations of MCP-1 in FF from women with tubal factor infertility may indicate chronic inflammatory changes, potentially leading to decreased fertilization rates (Xu *et al.*, 2006). MCP-1 is abnormally elevated in the FF of obese women and is negatively correlated with pregnancy rates in infertile women undergoing IVF (Buyuk *et al.*, 2017).

Sialic acid-binding immunoglobulin superfamily lectins (Siglec-11) expressed by human ovarian stromal fibroblasts interact with its ligands on mast cells, stimulating histamine secretion before ovulation and contributing to the inflammatory reaction during ovulation (Wang *et al.*, 2011). Interestingly, there is a trend of increased Siglec-11 expression in both postmenopausal and PCOS ovaries, which share some features, such as perturbed follicle growth and fertility deficiency, indicating potential roles for Siglec-11 in ovarian physiology (Wang *et al.*, 2011).

Furthermore, ovarian stromal cells are responsible for secreting ECM proteins, as well as MMPs and tissue inhibitors of MMPs (TIMPs), to facilitate maintenance and remodeling of the ovarian ECM (Briley *et al.*, 2016; Kinnear *et al.*, 2020). Mass spectrometry analysis of human FF stromal cells revealed the presence of 97 proteins associated with the stress response, positive regulation of apoptotic cell clearance, and embryo implantation (Skliutė *et al.*, 2023).

Ovarian stromal cells and cytokines are pivotal to ovarian physiology, and their aberrations are linked to IVF failure, POF, and PCOS. Nonetheless, owing to the constraints of present research, a thorough and exhaustive delineation of the contribution of stromal cells to ovarian functionality remains absent.

### Ovarian vascular system

The ovarian vasculature mainly consists of blood vessels and lymphatic vessels. The ovarian blood vessel system begins with proliferation and extension of the branches of the primordial gonadal vasculature at ∼11.5 days postcoitus (dpc) in mice (Coveney *et al.*, 2008). In human, the ovarian medulla typically contains larger blood vessels, including spiraling arteries and arterioles. At the cortico-medullary junction, small medullary arteries branch to cortical arterioles. Ovarian blood vessels play essential roles in providing oxygenation, hormone trafficking, nutrients, and facilitating waste removal. Lymphatic vessels develop postnatally, first becoming apparent in the hilus (stalk) of the ovary at postnatal day (P) 8.5 in mice (Brown *et al.*, 2010). These vessels extend from the ovarian medulla into the cortex adjacent to developing follicles and are closely associated with blood vessels (Brown and Russell, 2014). The lymphatic system functions to return extravascular fluid and proteins back to the bloodstream and participates in immune cell trafficking.

#### Ovarian blood vessels

Blood vessels play critical roles in processes such as tissue oxygenation, metabolism, and immune surveillance as a versatile transport network. Angiogenesis is involved in human folliculogenesis, ovulation, and corpus luteum formation (Devesa and Caicedo, 2019; Tomita, 2021). Resident primordial and early growing follicles do not possess an independent vascular network, therefore mammal ovarian stromal vessels are critical for maintenance of the resting stage of primordial follicles and the growth of primary follicles (Delgado-Rosas *et al.*, 2009; Gao *et al.*, 2013). During follicle maturation, the mouse vascular sheath forms two concentric vascular networks in the theca interna and the theca externa (Park *et al.*, 2022). Maintaining follicular vasculature and ensuring adequate blood supply to follicles are essential for establishing mammalian follicular dominance and the corpus luteum (McFee *et al.*, 2012; Fellus-Alyagor *et al.*, 2021). The moderate permeability observed during angiogenesis of dominance follicles and the corpus luteum may indicate a rapid stabilization of forming vessels, conferring a functional advantage by facilitating efficiency of hormone transmission (Fellus-Alyagor *et al.*, 2021). Additionally, reports on vascular changes in human corpus luteum have shown that angiogenesis is actively occurring during the early luteal phase and is completed by the midluteal phase, and that blood vessels decrease in number during the late luteal phase (Sugino *et al.*, 2005). Human follicle survival relies on ovarian angiogenesis; follicular atresia or death occurs when vascular endothelial cells are damaged or when the capillary network within the sheath layer is inadequately formed (Akiyama *et al.*, 2014).

Local changes in ovarian blood flow are intricately linked to alterations in the biosynthesis of prostaglandins and steroids and local factors such as IGFs and oxygen tension, which likely modulate ovarian angiogenic processes (Stouffer *et al.*, 2001). Additionally, angiogenic factors, such as members of the vascular endothelial growth factor (VEGF), angiopoietin (ANGPT), fibroblast growth factor 2 (FGF-2), and platelet-derived growth factor (PDGF), have been shown to stimulate endothelial proliferation, migration, and tube formation in the ovary. VEGF promotes an increase in ovarian microvascular permeability, providing nutrients for the development and growth of primary follicles, and facilitating ovulation of dominant follicles. Throughout ovarian follicular development, VEGF isoforms may act in an autocrine manner to stimulate the proliferation and survival of mammalian granulosa cells, or in a paracrine fashion to promote angiogenesis in the theca cells (Rosales-Torres *et al.*, 2010; Zamora-Gutierrez *et al.*, 2019). Conversely, a decrease in the expression of VEGF mRNA in sheep granulosa cells had been reported at the onset of follicular atresia (Rosales-Torres *et al.*, 2010). During cow corpus luteum development, the VEGF system created a proangiogenic milieu, which translates to an antiangiogenic state at the time of luteolysis (Guzman *et al.*, 2015). Treatment with VEGF antagonist or overexpression of VEGF antiangiogenic members has been shown to impair crow and mouse follicle progression, induce follicular atresia, hinder ovulation, and weaken subsequent luteal function (Qiu *et al.*, 2012; Zamora-Gutierrez *et al.*, 2019). Besides, graft incubation with VEGF isoforms had been demonstrated to preserve primary ovarian follicles and enhance angiogenesis in a human xenograft model (Wang *et al.*, 2013).

The density of blood vessels and factors related to angiogenesis are closely associated with ovarian pathological state. Ovaries from women with PCOS showed a 2-fold increase in blood vessel density in the superficial ovarian cortical stroma compared to age-matched controls. Increased vascularization of the superficial cortical stroma in PCOS ovaries can impact the cortical metabolic rate, thereby affecting the survival of primordial follicles and leading to early follicular growth (Delgado-Rosas *et al.*, 2009). Additionally, elevated levels of VEGF in FF had been reported in women with PCOS, suggesting a role for the growth factor in the stromal hypervascularity observed in this syndrome (Artini *et al.*, 2006; Patil *et al.*, 2021). Furthermore, a genome-wide association study identified a significant association between reduced VEGF and increased risk of POI, indicating VEGF may serve as an early biomarker for predicting POI (Wang *et al.*, 2023). VEGF increases vascular permeability and is essential for the process of angiogenesis during embryo implantation in women undergoing IVF treatment (Benkhalifa *et al.*, 2021; Wu *et al.*, 2021), while VEGF polymorphisms may disturb angiogenesis during invasion of the blastocyst, resulting in implantation failure and recurrent implantation failure (Elmi *et al.*, 2023; Mrozikiewicz *et al.*, 2023).

#### Ovarian lymphatic vessels

Lymphatic vessels regulate interstitial fluid content and transport cells to the immune system. Lymphatic networks have been detected in the ovaries of human, primates, rats, mice, pigs, ewes, and rabbits (Brown and Russell, 2014). Mammalian lymphatic vessels are found within the stromal compartment, in the cortex, and surrounding growing follicles (Svingen *et al.*, 2012; Tomita, 2021). Brown *et al.* (2010) found an abnormal phenotype of follicular development in a mouse model with lymphatic defects (Brown *et al.*, 2010). They revealed that mouse ovarian lymphatic vessel development lagged behind embryonic gonad vascular development, suggesting that lymphatic function only plays a role in the late stage of follicular maturation. Furthermore, during luteolysis, the bovine ovarian lymphatic system transports luteal cells out and participates in luteal degeneration (Abe *et al.*, 2014).

The VEGFC/VEGF receptor 3 (VEGFR3) signaling pathway is the primary mechanism regulating lymphangiogenesis. VEGFR3 is predominantly expressed on lymphatic endothelial cells, where it is instrumental in orchestrating the formation and morphological evolution of ovarian lymphatic vessels (Brown and Russell, 2014). In a mouse model, VEGFR3 antagonists effectively prevented lymphangiogenesis of mature follicles within ovaries, with fewer secondary follicles, a reduced pregnancy rate, retarded fetal growth, and increased abortion (Rutkowski *et al.*, 2013).

The precise function of the ovarian lymphatic system remains elusive, yet it likely plays a pivotal role in maintaining fluid balance and facilitating hormone transport. Alongside the vascular system, it contributes to the regulation of various disorders linked to ovarian fluid imbalance, such as PCOS, ovarian hyperstimulation syndrome, and massive ovarian oedema. A more comprehensive understanding of the structure and function of lymphatic vessels within human ovaries could shed light on whether lymphatics are further involved in the pathogenesis of ovarian diseases. Thorough investigations into the regulation and function of ovarian lymphatic systems in animal models will offer valuable insights for advancing research into the functional or spatial deficiencies associated with the aforementioned clinical ovarian diseases.

### Ovarian immune microenvironment

Approximately 40 years ago, an interaction between the immune system and ovarian function was proposed. The development of the ovary is blocked by removing the thymus, a critical immune organ. Immune cells present in ovaries include three main types: macrophages, T lymphocytes, and B lymphocytes. They are primarily distributed in the immature or static ovarian stroma, particularly in proximity to ovarian blood vessels. These cells, along with inflammatory factors, play pivotal roles in various fertility-related processes within ovaries, ranging from follicle development to ovulation and corpus luteum formation and regression.

#### Macrophages

The predominant immune cell type in ovaries is the macrophages. Studies conducted in human or animal have revealed a widespread presence throughout various ovarian processes, including follicle development, ovulation, corpus luteum formation and regression, and follicle atresia (Tang *et al.*, 2023). Recently, using high-dimensional single-cell mass cytometry, five macrophage populations have been identified in adult mice ovaries (Jokela *et al.*, 2020). Additionally, single-cell RNA sequencing of human ovaries identified four subtypes of ovarian macrophages, shedding light on their roles in orchestrating diverse immune response within the ovarian microenvironment as individuals age (Zhou *et al.*, 2024). In ovarian research, particular emphasis is placed on investigating the pro-inflammatory M1 type and tissue-remodeling M2 type macrophages.

Ovarian macrophages are involved in the activation and development of follicles. In bovine ovaries, macrophages had been found to regulate the nuclear factor κB (NF-κB) pathway, thereby participating in the process of primordial follicle selection (Liu *et al.*, 2009). Co-culture studies involving newborn mouse ovaries with different macrophage subtypes demonstrated that M1 macrophages activated primordial follicles by upregulating the PI3K/AKT/rapamycin (mTOR) pathway, while M2 macrophages inhibited primordial follicles by downregulating this pathway (Xiao *et al.*, 2022). Additionally, to investigate the role of M1 and M2 macrophages in folliculogenesis, researchers created M1-like CD11c DTR mice (CD11c depletion mice) and M2-like CD206 DTR mice (CD206 depletion mice) (Ono *et al.*, 2018). Compared to wild-type mice, folliculogenesis was impaired in CD11c DTR mice, while it remained normal in CD206 DTR mice, indicating the necessity of M1 populations in folliculogenesis (Ono *et al.*, 2018). Moreover, co-culturing macrophages with primary and early secondary mouse follicles or rat granulosa cells significantly enhanced follicle growth, survival, and granulosa cell proliferation (Tingen *et al.*, 2011). However, aberrant macrophage infiltration in the mouse ovary, induced by endogenous or exogenous factors, increased inflammatory potential, and disrupted folliculogenesis (Thornton *et al.*, 2020; Saccon *et al.*, 2022).

Macrophages also play a role in ovulation and postovulatory repair processes. Mice lacking colony-stimulating factor-1 (CSF-1) experience depleted populations of macrophages in various tissues, leading to prolonged oestrous cycles and reduced ovulation rates in mice (Cohen *et al.*, 2002). Consistent with this, study employing clodronate liposomes to deplete ovarian macrophages showed a significant decrease in ovulation rate of mice or rats (Van der Hoek *et al.*, 2000). Recently, single-cell investigations of the follicular microenvironment surrounding the metaphase II oocyte in human preovulatory follicles have revealed the infiltration of five clusters of macrophages near granulosa cells, highlighting their regulatory role in the subsequent ovulation process (Wu *et al.*, 2022). As ovulation initiates, the increase in LH promotes granulosa cell synthesis of numerous chemoattractants to recruit macrophages, such as MCP-1 (Bersinger *et al.*, 2014), C–C-motif ligand-20 (CCL-20) (Al-Alem *et al.*, 2015), and IL-1 (Dang *et al.*, 2017). Following ovulation, macrophages migrate to the developing corpus luteum, participating in its formation, steroid hormone secretion, and remodeling postdegeneration. Histological studies of human ovaries had shown that corpus luteum macrophages underwent numerical alterations during the menstrual cycle, increasing toward the end of the early luteal phase, remaining relatively stable during the midluteal phase, and decreasing in the late luteal phase (Gaytan *et al.*, 1998). An acute macrophage depletion CD11b-DTR mouse model had revealed the critical role of macrophages in supporting the extensive vascular network necessary for corpus luteum integrity and progesterone (P4) production (Turner *et al.*, 2011; Care *et al.*, 2013). In comparison, mice with transforming growth factor-beta1 (TGF-β1) null mutations, a key factor promoting macrophage alternative activation (M2), produced ∼75% less P4 in early pregnancy (Ingman *et al.*, 2006). In the late luteal phase, macrophages produce tumor necrosis factor-alpha (TNF-α), regulating prostaglandin F2α (PGF2α) synthesis to trigger mouse ovarian luteolysis (Care *et al.*, 2013).

Some pathological conditions of the ovaries can lead to infiltration and functional alterations of macrophages. PCOS is characterized by systematic, chronic, low-grade inflammation, and these patients commonly exhibit an increased number of macrophages in ovarian tissues (Xiong *et al.*, 2011; Rudnicka *et al.*, 2021). In a PCOS rat model induced by prolonged exposure to 5α-dihydrotestosterone, there was an increase in the ratio of M1 macrophages in antral and preovulatory follicles, which was associated with upregulated expression of the pro-inflammatory adipokine chemerin (Lima *et al.*, 2018). When granulosa cells were co-cultured with macrophages or macrophage secretions from PCOS rat ovaries, there was an increase in granulosa cell apoptosis (Figueroa *et al.*, 2015; Lima *et al.*, 2018).

#### Lymphocytes

Ovarian lymphocytes are classified into B and T lymphocytes, primarily found in ovarian medulla, FF, and corpus luteum (Bukulmez and Arici, 2000). B lymphocytes are exceptionally rare in ovary and are occasionally noted in the luteal tissue. T lymphocytes are generally absent in developing follicles but proliferate significantly after ovulation, primarily participating in ovulation, luteal formation, and luteal degeneration. In buffalo ovarian tissue, T lymphocytes contribute to ovulation by releasing bioactive cytokines (Ramadan *et al.*, 2001). Following follicle rupture, the neovascularization of the developing corpus luteum provides opportunities for direct interaction between luteal cells and resident or migratory immune cells. Specifically, CD8+ T lymphocytes can directly or indirectly communicate with human luteal cells during corpus luteum formation and luteolysis (Walusimbi and Pate, 2013).

In infertile patients with DOR, the FF exhibited an increased number and heightened function of effector CD8+ T lymphocytes compared to infertile patients with normal ovarian reserve (Zhao *et al.*, 2022). These CD8+ T lymphocytes secreted immune cytokine interferon gamma (IFN-γ), inducing granulosa cell apoptosis and exacerbating the progression of DOR. The dysregulation of T lymphocytes is implicated in the pathogenesis of autoimmune ovarian injury, as evidenced by the infiltration of CD3+ T lymphocytes around ovaries follicles in patients with autoimmune oophoritis (Bakalov *et al.*, 2005). CD4+Foxp3+ regulatory T (Treg) cells play a key role in maintaining peripheral immune tolerance and contribute to ovarian immune homeostasis by preventing T helper 1 (TH1)-like inflammation. Jiao *et al.* (2021) discovered a correlation between the absence of Treg cells and the severity of POI, with Treg cells effectively reversing TH1-mediated ovarian insufficiency in mice (Jiao *et al.*, 2021). Additionally, a recent study has revealed an increased production of TH1 cytokines (IFN-γ, TNF-α, IL-2) in FF lymphocytes of patients with PCOS compared to normal, suggesting the potential involvement of TH1 dominance in ovarian immune pathogenesis in patients with PCOS (Qin *et al.*, 2016). However, another study observed significantly higher levels of IL-13, a TH2 cytokine, and reduced levels of IL-12, a TH1 cytokine, in patients with PCOS than in normally ovulating women (Gallinelli *et al.*, 2003). Moreover, Li *et al.* (2019) found increased expression of programmed death 1 (PD-1) and decreased expression of IFN-γ in CD4+ T and CD8+ T cells in FF of infertile patients with PCOS (Li *et al.*, 2019). These discrepancies may arise from variations in study populations and analytical methods.

#### Immune cytokines

Immune cytokines exert diverse roles in follicular development and the maintenance of ovarian function, which is potentially influenced by the type, concentration, and local milieu of cytokines. TNF-α, a proinflammatory cytokine primarily produced by monocytes, is expressed in the ovarian tissues across various species (Faustman and Davis, 2010). TNF-α participates in regulating of gonadotrophin-induced steroid hormone production, granulosa cell proliferation, and differentiation, as well as ovulation and luteal function maintenance (Crespo *et al.*, 2010; Glister *et al.*, 2014; Samir *et al.*, 2017). Some studies have explored the association between FF TNF‐α and IVF outcomes. Elevated levels of TNF‐α in patient FF were associated with poor-quality oocytes, leading to reduced rates of fertilization, embryonic development, and pregnancy outcome (Wyse *et al.*, 2021). Blocking TNF‐α had been shown to improve implantation, clinical pregnancy, and live birth rates in young infertile women (Winger *et al.*, 2009). PCOS shares similar properties with many chronic inflammatory disorders, and elevated TNF-α levels had been observed in patients with PCOS (Artimani *et al.*, 2018). Furthermore, increased FF TNF‐α levels in women with PCOS were significantly and inversely correlated to FF 17β-estradiol (E2) levels (Amato *et al.*, 2003). A study exploring the relation between TNF-α polymorphisms and PCOS susceptibility suggested that TNF-α polymorphisms might influence the risk of developing PCOS in the overall population (Zhang *et al.*, 2020).

IL-1 is synthesized by activated mononuclear macrophages. The two primary prototypic cytokines in this family, IL-1α and IL-1β, are known to trigger the expression of various proinflammatory genes and are implicated in processes such as ovulation, oocyte maturation, and ovarian steroidogenesis (Silva *et al.*, 2020; Wan *et al.*, 2023). However, uncontrolled inflammation has adverse effects on ovarian function. Studies on IL-1α knockout mice revealed a higher pregnancy rate and increased litter size compared to wild-type mice, with these effects appearing from 2.5 months of age and persisting into advanced age (Uri-Belapolsky *et al.*, 2014). Furthermore, polymorphisms in IL-1α and IL-1β gene were found to be more prevalent in the PCOS group, increasing the risk of PCOS (Zhang *et al.*, 2020).

IL-6, predominantly secreted by monocytes/macrophages and granulosa cells under normal ovarian physiological conditions, acts as a powerful autocrine modulator of granulosa cell differentiation, ovarian cumulus cell functionality, COC expansion, and oocyte competence (Liu *et al.*, 2009; Imai *et al.*, 2014). However, IL-6 adversely impacted human and rat ovarian function by inhibiting FSH-stimulated E2 and P4 production in granulosa cells (Tamura *et al.*, 2000; Salmassi *et al.*, 2001). Elevated FF concentrations of IL-6 in patients with PCOS, as compared to non-PCOS women, indicate a significantly enhanced proinflammatory environment in PCOS FF (Kim *et al.*, 2011; Zhang *et al.*, 2017). Additionally, the IL-6-174 G/C polymorphism had been identified as a potential genetic indicator for PCOS susceptibility (Benjamin *et al.*, 2020; Azeez *et al.*, 2022). Notably, a high FF level of IL-6 was associated with improved clinical pregnancy rates and reduced embryo fragmentation during IVF treatments (Yang *et al.*, 2020; Stojanovic Gavrilovic *et al.*, 2022).

IFN-γ belongs to the interferon family that is produced by T lymphocytes, macrophages, and natural killer (NK) cells. IFN-γ regulates ovarian function by influencing granulosa differentiation, follicular atresia, steroid hormone synthesis, and luteal degeneration (Lee *et al.*, 2016; Wei *et al.*, 2022). In infertile patients with DOR, the level of IFN-γ in FF was found to be elevated, indicating a shift in the ovarian immune balance (Zhao *et al.*, 2022). Conversely, infertile patients with PCOS demonstrated significantly decreased expression of IFN-γ in FF compared to women with normal ovulation (Li *et al.*, 2019).

TGF-β1, mainly secreted by macrophages and lymphocytes, stands as a pivotal growth factor orchestrating diverse processes, including follicular development, steroidogenesis, ovulation, oocyte maturation, and luteinization (Cheng *et al.*, 2021; Guo *et al.*, 2022). In TGF-β1 null mutant mice, ovarian function was profoundly compromised with prolonged ovarian cycles, erratic ovulation, and a notable 40% decrease in ovulated oocytes (Ingman *et al.*, 2006). Furthermore, hypomethylation of CpG4 and CpG7 sites in the TGF-β1 gene promoter strongly correlated with the pathogenesis of insulin resistance-associated PCOS by modulating TGF-β1 gene expression (Gao *et al.*, 2024). Notably, individuals with PCOS exhibited an elevated TGF-β1 level, and the TGF-β1/SMAD3 signaling pathway inhibited ovarian follicular development by inducing granulosa cell apoptosis (Shen and Wang, 2019). Moreover, certain SNPs within the TGF-β1 gene, such as rs11466313, rs1800469, rs2317130, and rs4803457, were associated with PCOS susceptibility and phenotypic traits in Korean women (Roh *et al.*, 2017). Among these variants, the rs4803457 polymorphism emerged as a pivotal determinant in the pathogenesis of PCOS among Chinese Han women (Yang *et al.*, 2015).

### Ovarian stem cells

For many years, ovarian biology has been based on the doctrine that the oocyte reserve of female mammals is determined by the quantity and quality of the primordial follicle pool that developed during the neonatal period, and that ovarian follicular reserve is generally lost with age, without renewal (Johnson *et al.*, 2004). However, in recent years, proponents of neo-oogenesis have argued for the existence of renewable germ stem cells in mammalian ovaries that are capable of differentiating into oocytes (Gheorghisan-Galateanu *et al.*, 2014).

Johnson *et al.* (2005) made a groundbreaking discovery, revealing the presence of mitotically active germ cells in mouse ovaries after birth, suggesting a potential origin from bone marrow (Johnson *et al.*, 2005). Subsequently, germline stem cells from mouse ovaries were successfully isolated and cultured (Zou *et al.*, 2009). When transplanted into the ovaries of chemotherapy-induced infertile mice, these germline stem cells differentiated into mature eggs, leading to the birth of offspring. Furthermore, germline stem cells were identified in the ovaries of women of reproductive age (White *et al.*, 2012). Since then, various research groups have reported the presence of germline stem cell populations in the ovaries of numerous mammal species (White *et al.*, 2012; Stimpfel *et al.*, 2013; Woods and Tilly, 2013; Dunlop *et al.*, 2014; Clarkson *et al.*, 2018; Silvestris *et al.*, 2018; Sharma and Bhartiya, 2022). Interestingly, our group recently isolates DEAD-box helicase 4 (DDX4+) germline stem cells from the ovaries of postmenopausal women, demonstrating their capability to enter the meiosis stage and differentiate into oocytes (Wu *et al.*, 2022). Nevertheless, the existence and significance of ovarian germline stem cells remain contentious. Lei and Spradling (2013) failed to detect active germline stem cells in mouse ovaries using a sensitive lineage-labeling system (Lei and Spradling, 2013). Similarly, Wagner *et al.* (2020) found that the cells captured by DDX4 antibodies in the human ovarian cortex were perivascular cells rather than oogonial stem cells (Wagner *et al.*, 2020). Therefore, much remains to be elucidated regarding the biology of ovarian stem cells and their potential clinical applications.

## Changes in the ovarian microenvironment during chemotherapy

**Figure 2.**
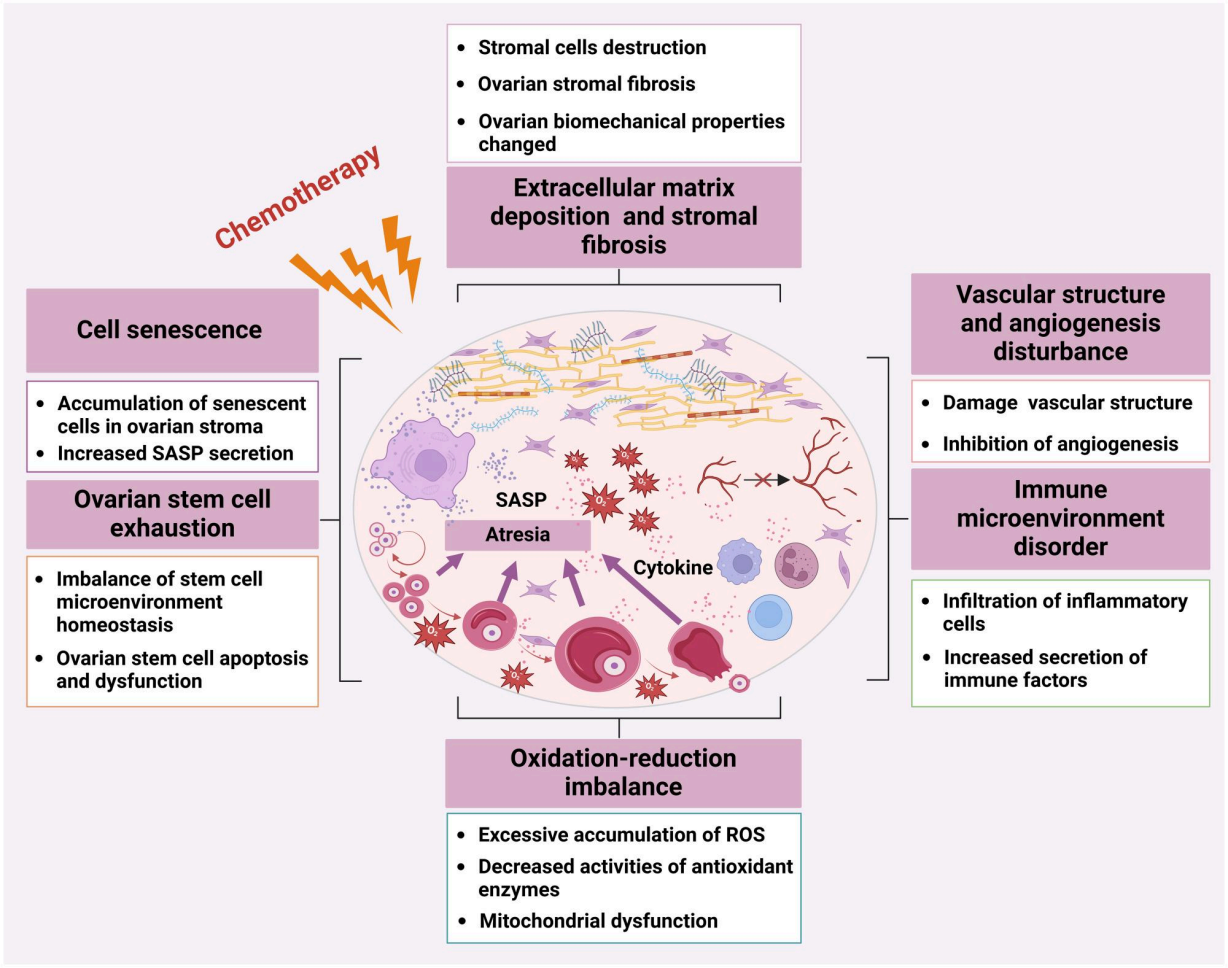
**Changes in the ovarian microenvironment caused by chemotherapy.** ROS, reactive oxygen species; SASP, senescence-associated secretion phenotype. Created with BioRender.com, with permission.

In addition to directly inducing follicle apoptosis, chemotherapeutic agents can indirectly affect ovarian function by damaging the ovarian microenvironment. The ovarian microenvironment shows great changes during chemotherapy, inducing ECM deposition and stromal fibrosis, disordered angiogenesis, immune microenvironment disturbance, oxidative stress homeostasis imbalance, ovarian stem cell exhaustion, and cell senescence (Fig. 2; Table 1). The homeostatic imbalance of the microenvironment induced by chemotherapy will lead to ovarian dysfunction and eventually accelerate ovarian ageing.

**Table 1.** Damage caused to the ovarian microenvironment by chemotherapy drugs.

Microenvironment damage	Drug	Model	Finding	Reference
Extracellular matrix deposition and stromal fibrosis	Alkylating agents	Human	Ovarian tissue had been replaced by collagenous connective tissue.	Meirow *et al.*, 2007
	Alkylating agents and anthracyclines	Human	A higher content of collagen and DNA damage was observed in ovarian stromal.	Pampanini *et al.*, 2019; Shai *et al.*, 2021
	CTX	Rabbit	Increased collagen fibers.	Abd-Allah *et al.*, 2013
	CTX	Rat	Hyperplasia was detected in ovarian stromal cells.	Abdel-Raheem *et al.*, 2015
	CTX	Mouse	The atrophied ovaries were mainly composed of stromal cells.	Liu *et al.*, 2012; Zhu *et al.*, 2015; Huang *et al.*, 2023
	CTX	Mouse	Increased ovarian fibrotic area.	Chen *et al.*, 2021
	CTX	Rat	Induced ovarian stromal fibrosis and luteal fibrosis.	Huang *et al.*, 2009; Abdelzaher *et al.*, 2021
	CTX	Rat	The tensile mechanical properties of the ovarian tissue reduced.	Pan *et al.*, 2017
	CIS	Rat	Fibrosis was detected in ovarian stroma.	Atli *et al.*, 2017; Chinwe *et al.*, 2018; Said *et al.*, 2019; Ciplak *et al.*, 2023
	CIS	Rat	Led to excessive synthesis of ovarian ECM.	Cui *et al.*, 2020
	CIS	Rat	The expression of Col-I, Col-III, fibronectin, CTGF, and α-SMA increased.	Cui *et al.*, 2022
	DOX	Mouse	Induced an acute insult of ovarian parenchymal fibrosis.	Ben-Aharon *et al.*, 2010
	DOX	Mouse	The expression of α-SMA, Col-I, TGF-β1, TIMP1, TIMP2, and MMP2 increased.	Gao *et al.*, 2023
	CTX; PTX; DOX; CIS	Mouse	Ovarian stromal disorder and atrophy.	Zhang *et al.*, 2023
	CIS; DOX	Human	The growth of ovarian stromal cell was inhibited.	Fabbri *et al.*, 2016; Lopes *et al.*, 2020
	CIS; DOX	Mouse	The growth inhibition and apoptosis of ovarian stromal cell were induced.	Roti Roti *et al.*, 2012; Yoon *et al.*, 2020
	CTX/BUL	Mouse	Structural disruption of the ovarian interstitial region.	Buigues *et al.*, 2020
	CTX/BUL	Mouse	Severe ovarian interstitial fibrosis.	Luo *et al.*, 2017; Yang *et al.*, 2019
	Docetaxel/CTX	Rat	The expression of TGF-β1, Col-I, and Col-III was increased.	De Moraes *et al.*, 2016
Vascular structure and angiogenesis disturbance	Alkylating/anthracycline/taxane agents	Human	The narrowing and obliteration of ovarian blood vessels, decreased vessel area, reduced blood flow, and occasional neovascularization.	Nicosia *et al.*, 1985; Marcello *et al.*, 1990; Meirow *et al.*, 2007; Ben-Aharon *et al.*, 2012; Shai *et al.*, 2021; Devos *et al.*, 2023
	CTX; CIS; PTX	Human	The vascular structure was sparse and the microvascular density decreased.	Bildik *et al.*, 2015
	CTX	Rabbit	Vascular smooth muscle proliferation and vascular wall thickening.	Abd-Allah *et al.*, 2013
	CTX	Rat	Thickening of the ovarian tissue vascular wall and hyaluronic degeneration.	Fu *et al.*, 2017
	CTX	Mouse	Ovarian microvascularization was impaired. Reduced the expression of VEGF.	Ezoe *et al.*, 2014; Dynes *et al.*, 2017; Oubina *et al.*, 2021; Huang *et al.*, 2023
	CTX/BUL	Mouse	The ovarian vascularization area was decreased. The expression of vWF, IGF-1, ANGPT, and CD34 decreased.	Herraiz *et al.*, 2018; Yang *et al.*, 2019; Buigues *et al.*, 2020; Huang *et al.*, 2021; Salvatore *et al.*, 2021
	CIS	Rat	Damaged ovarian vascular structure.	Dayangan Sayan *et al.*, 2018; Said *et al.*, 2019; Ciplak *et al.*, 2023
	DOX	Mouse	Disorganized immature ovarian blood vessels with areas of discontinuation in the endothelial layer. The expression of CD34 and VEGF decreased.	Herrero *et al.*, 2023
	CTX	Mouse	The mRNA expression of VEGF and IGF-1 decreased.	Eslami *et al.*, 2023
	CTX	Mouse	Reduced the expression of VEGF, bFGF, PDGFB, and ANG.	Abdelzaher *et al.*, 2021; Zhou *et al.*, 2021
	CIS	Mouse	The expression of VEGF, IGF-1, FGF, and CD31 was downregulated.	Qu *et al.*, 2022
Immune microenvironment disorder	CTX	Human; Rhesus; Macaque	The clusters of CD68/CD163+ macrophages, CD4+ lymphocytes, CD3+ T lymphocytes, and MPO-positive neutrophils were increased.	Du *et al.*, 2022
	CTX	Mouse	Increased infiltration of M0 macrophages, naive B cells, resting NK cells, and reduced infiltration of Treg cells, TH17 cells, NK cells, and M1/M2 macrophages ratio.	He *et al.*, 2023
	CTX/BUL	Mouse	Macrophages and neutrophils infiltrated in ovary.	Deng *et al.*, 2021
	CIS	Rat	The infiltration of neutrophils increased.	Dinc *et al.*, 2023
	CIS	Rat	Led to leukocyte accumulation and elevation of MPO.	Atli *et al.*, 2017; Mentese *et al.*, 2022; Ciplak *et al.*, 2023
	CTX	Mouse	Decreased the level of IL-2 and TNF-α.	Liu *et al.*, 2019
	CTX	Mouse	Increased the expression of IL-6, IL-8, and TNF-α, as well as decreased the expression of IL-10 and TSG-6.	Deng *et al.*, 2021; Eslami *et al.*, 2023
	CTX	Rat	Increased the expression of IL-6, IL-1β, and TNF-α and decreased the expression of IL-10.	Abdel-Raheem *et al.*, 2015; Gabr *et al.*, 2016; Ling *et al.*, 2017; Hassan *et al.*, 2021
	CTX/BUL	Mouse	Downregulated IL-2 and TNF-α, and upregulated IL-4.	Huang *et al.*, 2021; Li *et al.*, 2023
	CTX/BUL	Mouse	Increased the expression of TNF-α, IL-8, and IL-6.	Lai *et al.*, 2014
	CIS	Rat	The levels of TNF-α, IL-1β, NF-kB, and IL-6 were increased.	Said *et al.*, 2019; Ibrahim *et al.*, 2021; Al-Shahat *et al.*, 2022; Mentese *et al.*, 2022; Dinc *et al.*, 2023
	DOX	Human	The level of TNF-α, COX-2, IL-6, IL-8, MMP2, and MMP9 was increased.	Fabbri *et al.*, 2019
Oxidation-reduction imbalance	CTX	Mouse	Increased the expression of MDA, LDH and decreased the expression of GPx, CAT, and SOD.	Huang *et al.*, 2019, 2023; Ding *et al.*, 2020; Khanmohammadi *et al.*, 2021; Feng *et al.*, 2022; Zhao *et al.*, 2022
	CTX	Rat	Increased the expression of MDA and decreased the expression of GSH, GPx, CAT, and SOD.	Abdel-Raheem *et al.*, 2015; Khedr, 2015; Saleh and Mansour, 2016; Yang *et al.*, 2017; Abdelzaher *et al.*, 2021; Hassan *et al.*, 2021; Talebi *et al.*, 2022; Zheng *et al.*, 2022
	CTX	Mouse	Decreased ATP and mtDNA production and increased mitochondrial membrane potential reduction rate.	Chen *et al.*, 2022
	CTX	Mouse	The expression of 8-OHdG, NTY, and 4-HNE significantly increased.	Chen *et al.*, 2021
	CTX	Mouse	Enhanced antioxidant enzymes and decreased ROS and MDA.	Athira *et al.*, 2020
	CTX/BUL	Mouse	Induced excess ROS.	Chen *et al.*, 2021; Zhang *et al.*, 2021; Peng *et al.*, 2023
	CTX/BUL	Mouse	Impaired biogenesis of oocyte mitochondria and reduced expression of 8-OHdG, MDA, and PGC1α.	Dai *et al.*, 2022
	CIS	Rat	Decreased the levels of SOD, GSH, CAT, Cu/Zn-SOD, and increased the levels of oxidized MDA, NOx.	Li *et al.*, 2013; Meng *et al.*, 2015; Chinwe *et al.*, 2018; Soyman *et al.*, 2018; Ibrahim *et al.*, 2021; Al-Shahat *et al.*, 2022; Mentese *et al.*, 2022; Dinc *et al.*, 2023
	CIS	Mouse	Decreased the levels of CAT and GPx, and increased 4-HNE, NTY, 8-OHdG, and MDA.	Biyik *et al.*, 2021; Wang *et al.*, 2024
	CIS	Mouse	Increased the production of ROS and decreased the activity of mitochondria.	Chen *et al.*, 2015; Barberino *et al.*, 2017; Lins *et al.*, 2020; Gouveia *et al.*, 2021
	DOX	Mouse	Decreased the mRNA expression of SOD, GSH and increased the expression of oxidative stress-related genes, such as MDA and NRF2.	Niringiyumukiza *et al.*, 2019; Wang *et al.*, 2020
	DOX	Mouse	Increased the expression of HO-1 and CAT.	Herrero *et al.*, 2023
	DOX	Rat	Increased MDA and NO levels, decreased SOD level.	Morsi *et al.*, 2023
Ovarian stem cell exhaustion	CTX	Mouse	The expression of MVH and OCT4 was reduced.	Jiang *et al.*, 2019
	CTX	Mouse	Decreased the number of ovarian stem cells and diminished expression of MVH and OCT4.	Jiang *et al.*, 2019
	CTX/BUL	Mouse	Ovarian stem cell apoptosis and dysfunction.	Lai *et al.*, 2015; Sriraman *et al.*, 2015; Wu *et al.*, 2019.
	ABVD	Human	Biovular and binucleate follicles were presented in ovaries.	McLaughlin *et al.*, 2017
Cell senescence	CTX	Mouse	Induced ovarian granulosa cell senescence and stromal cell with higher expression of p53, p66Shc, and p16.	Xiong *et al.*, 2017; Ai *et al.*, 2023; Xu *et al.*, 2023
	CTX/BUL	Mouse	Senescent ovarian stromal cells increased and the expression of cell p53, p21, and p27 was upregulated.	Dai *et al.*, 2022
	CIS	Mouse	Increased cell senescence and the expression of SASPs (IL-6, IL-1β).	Marcozzi *et al.*, 2019; Du *et al.*, 2022
	DOX	Mouse	The accumulation of senescent cells and the expression of p16 and p21 increased. The expression of SASPs (IL-6, MCP-1, TGF-β1) increased.	Gao *et al.*, 2023

α-SMA, α-smooth muscle actin; ANG/ANGPT, angiopoietin; ABVD, adriamycin, bleomycin, vinblastine and dacarbazine; BUL, busulphan; CTX, cyclophosphamide; CIS, cisplatin; CAT, catalase; CD31, platelet endothelial cell adhesion molecule; CTGF, connective tissue growth factor; Col-I, type I collagen fiber; Col-III, type III collagen fiber; COX-2, cyclooxygenase-2; DOX, doxorubicin; ECM, extracellular matrix; FGF, fibroblast growth factor; GPx, lutathione peroxidase; GSH, glutathione; 8-OHdG, 8-Hydroxy-2′-deoxyguanosine; 4-HNE, 4-hydroxynonenal; HO-1, heme oxygenase-1; IGF-1, insulin-like growth factor-1; LDH, lactate dehydrogenase; MPO, myeloperoxidase positive; MDA, malondialdehyde; MMP2, metalloproteinase 2; MMP9, metalloproteinase 9; MCP-1, monocyte chemoattractant protein-1; MVH, mouse vasa homolog; NRF2, NF-E2-related factor 2; NTY, nitrotyrosine; NF-kB, nuclear factor kappa B; NO, nitric oxide; NOx, total nitric oxide; OCT4, octamer-binding transcription factor 4; PTX, paclitaxel; PDGFB, platelet-derived growth factor B; PGC1α, peroxisome proliferator-activated receptor gamma coactivator 1-alpha; ROS, reactive oxygen species; SOD, superoxide dismutase; SASPs, senescence-associated secretion phenotypes; Cu/Zn-SOD, Cu/Zn superoxide dismutase; TSG-6, TNF-stimulated gene 6; TNF-α, tumor necrosis factor-alpha; TGF-β1, transforming growth factor-beta1; TIMP1, metalloprotease 1; TIMP2, metalloprotease 2; vWF, von Willebrand factor; VEGF, vascular endothelial growth factor.

### Extracellular matrix deposition and stromal fibrosis

Under normal physiological conditions, tissues undergo remodeling in response to injury, leading to tissue regeneration without permanent damage. Conversely, fibrosis arises from repeated tissue insult and inflammation. Ovarian fibrosis is characterized by excessive ovarian stromal cell proliferation and ECM deposition and is one of the main causes of ovarian dysfunction (Zhou *et al.*, 2017). Fibrosis will destroy the normal ovarian physiological structure; if not corrected in time, fibrous connective tissue will replace functional tissue, and ovarian function will further decline or even fail, with loss of periodic ovulation and ovarian endocrine function (Briley *et al.*, 2016). Fibrosis in the ovarian stroma tends to increase with advanced reproductive age, although the precise mechanism remains unknown. One reason could be the compromised biomechanical properties of an aged ovary, such as increased synthesis and deposition of collagen, diminished hyaluronan levels, or alteration in posttranslational modifications (Amargant *et al.*, 2020; Babayev *et al.*, 2023; DipaLi *et al.*, 2023). Alternatively, an imbalance of the activities of MMPs and TIMPs disrupts the homeostasis of ECM synthesis and metabolism (Briley *et al.*, 2016). As the ovarian microenvironment or stroma ages, it tends to become fibro-inflammatory, characterized by increased production and release of proinflammatory and pro-fibrotic cytokines and growth factors, as well as a shift in macrophage populations towards multinucleated macrophage giant cells (Machlin *et al.*, 2021; Babayev *et al.*, 2023). Furthermore, one study revealed an accumulation of non-heme iron in the ovarian stroma with reproductive aging, indicating iron accumulation in the aging ovary may contribute to fibrosis (Asano, 2012).

Compared to ovarian fibrosis during natural aging, chemotherapy drugs such as CTX, CIS, and DOX have been implicated in disrupting the balance between the synthesis and degradation of ECM, leading to excessive ECM accumulation and fibrosis (Briley *et al.*, 2016; Zhou *et al.*, 2017). A study conducted on frozen sections of ovarian tissue from cancer survivors undergoing alkylating chemotherapy revealed the replacement of intact ovarian tissue with collagenous connective tissue, with no follicles observed in the fibrotic area (Meirow *et al.*, 2007). This suggests that chemotherapy causes structural damage to the ovary by promoting fibrosis of ovarian cortex, leading to focal loss of primordial follicles. Besides, a significant correlation has been found between the extent of ovarian fibrosis and the cumulative exposure to alkylating agents and anthracyclines in cancer patients (Pampanini *et al.*, 2019; Shai *et al.*, 2021), with fibrosis typically observed 4–6 months after exposure to alkylating agents (Shai *et al.*, 2021).

Animal studies had also shown that low-dose CTX administration for 2 weeks induced a significant increase in collagen fibers, absence of follicles in the fibrotic zone, and ovarian tissue atrophy in rabbits (Abd-Allah *et al.*, 2013). Moreover, hyperplasia was detected in rat ovarian stromal cells, with follicular atresia observed after CTX administration (Abdel-Raheem *et al.*, 2015). In a CTX-induced POF mouse model, atrophied ovaries primarily composed of stromal cells within a fibrous matrix were observed, accompanied by a reduced number of follicles at each stage (Liu *et al.*, 2012; Zhu *et al.*, 2015; Huang *et al.*, 2023). Histological staining of mouse ovaries post-CTX treatment had revealed that fibrotic areas are predominantly located in the interstitium between follicles and near the ovarian cortex (Chen *et al.*, 2021). Similarly, in a rat model, CTX induced ovarian stromal fibrosis, luteal fibrosis, and ovarian vacuolar degeneration (Huang *et al.*, 2009; Abdelzaher *et al.*, 2021). Ovarian fibrosis leads to changes in biomechanical properties, disrupting the normal follicular developmental environment. Parameters such as maximum load, maximum stress, maximum strain, elastic limit strain, elastic limit load, and elastic limit stress in rat ovaries subjected to CTX treatment were found to be lower than those in the normal control group, indicating changes in the tensile mechanical properties of ovarian tissue following the loss of structural integrity and chemotherapy-induced ovarian fibrosis (Pan *et al.*, 2017).

Following CIS therapy, fibrosis has been detected in rat ovarian stroma (Atli *et al.*, 2017; Chinwe *et al.*, 2018; Said *et al.*, 2019; Ciplak *et al.*, 2023). During mammalian follicular development, inner theca cells maintain the integrity of follicular structure, while outer myofibroblasts secrete ECM, including type I collagen fiber (Col-I) and type III collagen fiber (Col-III), facilitating the formation of follicular capillaries (Tingen *et al.*, 2011; Hummitzsch *et al.*, 2019; Kinnear *et al.*, 2020; Isola *et al.*, 2024). In CIS-induced POI rat models, ovarian stromal cells predominantly proliferated and differentiated into outer myofibroblasts, leading to excessive synthesis of ovarian ECM and subsequent ovarian fibrosis (Cui *et al.*, 2020). This process involves activation of the TGF-β1/SMAD3 signal pathway to promote the proliferation of ovarian fibroblasts. Cui *et al.* (2022) demonstrated that, following CIS treatment, rat ovarian tissue exhibited increased expression of fibrosis-related markers, such as Col-I, Col-III, fibronectin, connective tissue growth factor (CTGF), and alpha-smooth muscle actin (α-SMA) (Cui *et al.*, 2022). In addition, they discovered that CIS promoted the transformation of stromal cells into myofibroblasts by downregulating the expression of orphan nuclear receptor 4A1 (NR4A1) and increasing AMP-activated protein kinase phosphorylation.

DOX chemotherapy has been identified as a potential inducer of ovarian toxicity through promoting ovarian fibrosis. A study of mice subjected to DOX treatment revealed a reduction in ovarian size and weight, which may involve an acute insult of ovarian ischemia and parenchymal fibrosis (Ben-Aharon *et al.*, 2010). The mRNA expression levels of mouse α-SMA, Col-I, TGF-β1, TIMP1, TIMP2, and MMP2, which are involved in tissue remodeling and fibrosis progression, were significantly increased in mouse ovaries of DOX-treatment group (Gao *et al.*, 2023). Interestingly, mice treated with various chemotherapy agents, including CTX, paclitaxel, DOX, and CIS, exhibited ovarian stromal disorder and atrophy alongside significant upregulation of TGF-β. Among these agents, DOX treatment displays the highest toxicity towards ovarian stroma and caused the most pronounced fibrosis (Zhang *et al.*, 2023). Additionally, CIS and DOX could induce growth inhibition and apoptosis in ovarian stromal cells obtained from human and mouse ovarian tissue (Roti Roti *et al.*, 2012; Fabbri *et al.*, 2016; Lopes *et al.*, 2020; Yoon *et al.*, 2020).

Combinations of antitumor agents have long been recognized as a critical approach to treatment. Structural disruption within the ovarian interstitial region had been observed in DOR and POI mouse models induced by combined therapy with CTX and BUL (Buigues *et al.*, 2020). In these models, stromal degeneration was found to be increased by 2.5 times and 3.3 times in the DOR and POI models, respectively. Additionally, other studies had reported that CTX/BUL treatment induced severe ovarian interstitial fibrosis in a mouse model of POF/POI (Luo *et al.*, 2017; Yang *et al.*, 2019). Furthermore, De Moraes *et al.* (2016) assessed the effects of docetaxel combined with CTX (TC) on ovarian stromal tissue in rats (De Moraes *et al.*, 2016). Their findings revealed increased expression levels of TGF-β1, Col-I, and Col-III in ovarian tissue from the TC group. Moreover, the collagen fiber structure within the ovary was disorganized, suggesting induction of ovarian fibrosis by the TC regimen.

Based on the existing literature, we have summarized the possible mechanisms of ovarian fibrosis associated with chemotherapy. Following chemotherapy treatment, ovarian stromal cells proliferate and differentiate into myofibroblasts, which secrete various fibrosis-related factors (Col-I, Col-III, fibronectin, CTGF, α-SMA, etc) and excessively synthesize ECM, ultimately leading to focal fibrosis of the ovarian cortex. Additionally, ovarian fibrosis leads to a change in the tensile mechanical properties of ovarian tissue, disrupting the normal follicular developmental environment. Furthermore, our results on the changes in ovarian microenvironment during chemotherapy demonstrate that there is decreased vascular density, increased levels of pro-inflammatory cytokines, significant infiltration of immune cells, and excessive production of ROS. Thus, immune cells and pro-inflammatory cytokines may be involved in creating fibrotic regions of ovarian tissue, inducing ovarian stroma degeneration and atrophy. Oxidative stress and the anti-angiogenic effects of chemotherapy result in insufficient stromal nutrient supply and stromal cell damage, ultimately inducing stromal cell apoptosis and stromal degeneration.

### Vascular structure and angiogenesis disturbance

As chemotherapy is not selective, it affects both the tumor and the host’s healthy cells. This can lead to chemotherapy-induced vascular toxicity, characterized by direct or acute effects such as endothelial dysfunction, increased vascular muscle tone, and the constriction and distortion of blood vessels (Mizrachi *et al.*, 2021). Likewise, chemotherapy drugs inhibit ovarian angiogenesis and destroy the integrity and function of blood vessels, ultimately resulting in ovarian dysfunction.

Several studies had demonstrated the presence of ovarian vascular alterations in patients exposed to chemotherapy, including narrowing and obliteration of blood vessels, decreased vessel area, reduced blood flow, and occasional neovascularization (Nicosia *et al.*, 1985; Marcello *et al.*, 1990; Meirow *et al.*, 2007; Ben-Aharon *et al.*, 2012; Shai *et al.*, 2021; Devos *et al.*, 2023). Following chemotherapy, the human ovarian cortex exhibited sparse vascular structure, with microvascular density decreased to 18%, 22%, and 56% after treatment with CTX, CIS, and paclitaxel, respectively (Bildik *et al.*, 2015). CTX also induced vascular smooth muscle proliferation and thickening of vascular walls in rabbit ovaries, and resulted in blood vessel narrowing and obliteration (Abd-Allah *et al.*, 2013). Further investigations have linked chemotherapy-induced rabbit ovarian vascular toxicity to abnormal follicle development and ovarian dysfunction (Abd-Allah *et al.*, 2013). In another study, CTX treatment led to thickening of the ovarian tissue vascular wall and hyaluronic degeneration in rats (Fu *et al.*, 2017). Chemotherapy drugs can directly damage ovarian blood vessels, resulting in a decrease in vascular density. The vascularization area of the ovary was significantly decreased in a POF mouse model induced by CTX or CTX/BUL (Herraiz *et al.*, 2018; Buigues *et al.*, 2020; Huang *et al.*, 2021, 2023; Oubina *et al.*, 2021; Salvatore *et al.*, 2021). CIS treatment directly damaged the ovarian vascular structure in rats, leading to vascular congestion and bleeding, which hinders the normal blood supply to the ovary (Dayangan Sayan *et al.*, 2018; Said *et al.*, 2019; Ciplak *et al.*, 2023). DOX treatment also induced disorganized immature ovarian blood vessels with areas of discontinuation in the endothelial layer in mice (Herrero *et al.*, 2023).

Ovarian angiogenesis is a complex process involving various angiogenic factors, such as VEGF, IGF-1, FGF, ANGPT, and platelet-derived growth factor B (PDGFB). Chemotherapy drugs can interfere with the expression of genes related to angiogenesis and damage vascular endothelial cells, leading to ovarian angiogenesis disorders. Studies had shown that chemotherapy, such as CTX, suppressed the mRNA expression level of VEGF and IGF-1 in mouse ovary, resulting in inadequate vascular endothelial cell coverage and poor vascular maturation (Eslami *et al.*, 2023). Additionally, reduced protein expression of VEGF, FGF, PDGFB, and ANGPT was observed in the ovary of CTX-induced POF mouse and rat models (Abdelzaher *et al.*, 2021; Zhou *et al.*, 2021). These findings strongly suggest that CTX downregulates the transcription 3 (STAT3)/hypoxia-inducible factor-1 alpha-/VEGF signaling pathway, thus inhibiting ovarian angiogenesis. Long-term studies had demonstrated a positive correlation between CTX dosage and dysfunctional ovarian angiogenesis in mice, highlighting its lasting effects on VEGF expression (Ezoe *et al.*, 2014; Dynes *et al.*, 2017). Similarly, the expression of VEGF, IGF-1, FGF, and platelet endothelial cell adhesion molecule (CD31) were downregulated in ovary of CIS-treated mice compared with a control group (Qu *et al.*, 2022). Chemotherapy combination therapy with CTX/BUL reduced the expression of VEGF through inhibition of the PI3K/AKT pathway, and suppressed the expression of other angiogenic factors, such as von Willebrand factor (vWF), CD34, IGF-1, and ANGPT, ultimately inhibiting mouse ovarian angiogenesis (Herraiz *et al.*, 2018; Yang *et al.*, 2019; Buigues *et al.*, 2020; Huang *et al.*, 2021; Salvatore *et al.*, 2021). Furthermore, DOX treatment decreased CD34 and VEGF expression in mice, suggesting a decline in endothelial cells number (Herrero *et al.*, 2023). These finding collectively underscore the detrimental effects of chemotherapy on ovarian angiogenesis and highlight the importance of understanding and managing these effects in clinical practice.

### Immune microenvironment disorder

Homeostasis of the ovarian immune microenvironment is essential for the proper functioning of various physiological processes in the reproductive system, including follicle development, ovulation, and corpus luteum formation. Chemotherapy drugs have the potential to disrupt the delicate balance by triggering significant infiltration of inflammatory cells and the secretion of immune factors in the ovary, ultimately leading to disruption of the ovarian immune microenvironment.

In a clinical study, clusters of CD68/CD163+ macrophages, CD4+ lymphocytes, CD3+ T lymphocytes, and myeloperoxidase (MPO)-positive neutrophils were significantly increased in the ovarian cortex of cancer patients after chemotherapy (Du *et al.*, 2022). Additionally, macrophage infiltration into ovarian cortex was evident not only in humans and rhesus macaques but also in mouse ovarian cortex after CTX treatment. Prolonged treatment duration of up to 8 weeks resulted in macrophages invading follicles, leading to progressive ovarian tissue damage and follicle loss. This multi-species research indicates that chemotherapy-induced chronic inflammation can cause additional tissue damage to the ovary. In a mouse model receiving CTX treatment, immune cell analysis demonstrated increased infiltration of M0 macrophages, naive B cells, resting NK cells, and T cells in the ovaries, alongside reduced infiltration of Treg cells, TH17 cells, active NK cells, and altered M1/M2 macrophage ratio (He *et al.*, 2023). These results suggest a significant enhancement of inflammatory and immune responses in the ovaries of mice following chemotherapy. Similarly, in mice treated with CTX/BUL, numerous macrophages and neutrophils infiltrated the ovary, with macrophages primarily distributed in the corpus luteum and atretic follicle, while neutrophils were predominantly located around the corpus luteum and within follicles (Deng *et al.*, 2021). In a rat model of ovarian injury induced by CIS, the infiltration of neutrophils in the ovary led to structural disorder and degeneration of the developing follicle, along with local oedema of interstitial tissue and corpus luteum (Dinc *et al.*, 2023). CIS treatment led to leukocyte accumulation and elevated MPO levels, leading to rat ovarian tissue congestion, oedema, and follicular degeneration (Atli *et al.*, 2017; Mentese *et al.*, 2022; Ciplak *et al.*, 2023).

Alongside changes in ovarian immune cell populations, chemotherapy agents disrupt ovarian function by inducing an imbalance of inflammatory factors. In CTX-induced POF mice, ovarian levels of IL-2 and TNF-α decreased, likely attributed to reduced CD4+ T cells (Liu *et al.*, 2019). Additionally, increased expression of proinflammatory factors, IL-6, IL-8, and TNF-α, along with decreased expression of the anti-inflammatory factor IL-10 and TNF-stimulated gene 6 (TSG-6), were found in mouse ovaries after CTX treatment (Deng *et al.*, 2021; Eslami *et al.*, 2023). Similarly, rat ovaries showed increased expression of IL-6, IL-1β, and TNF-α, along with decreased expression of IL-10 after CTX treatment (Abdel-Raheem *et al.*, 2015; Gabr *et al.*, 2016; Ling *et al.*, 2017; Hassan *et al.*, 2021). CTX/BUL treatment decreased expression of IL-2 and TNF-α while increasing expression of IL-4 in mouse ovaries (Huang *et al.*, 2021; Li *et al.*, 2023). The disruption of the TH1/TH2 balance in the ovary by chemotherapy-induced changes in IL-2, TNF-α, and IL-4 suggests potential induction of ovarian dysfunction and immunosuppression. In another study, several proinflammatory cytokines, such as TNF-α, IL-8, and IL-6, were significantly increased in mouse ovaries that received CTX/BUL chemotherapy (Lai *et al.*, 2014). Tissue levels of the proinflammatory cytokines TNF-α, IL-1β, NF-κB, and IL-6 were also elevated in mouse and rat ovaries treated with CIS (Said *et al.*, 2019; Ibrahim *et al.*, 2021; Al-Shahat *et al.*, 2022; Mentese *et al.*, 2022; Dinc *et al.*, 2023). An *in vitro* study found a significant increase in the expression of pro-inflammatory cytokines (TNF-α, cyclooxygenase-2, IL-6, IL-8, MMP2, and MMP9) in human ovarian tissue exposed to DOX (Fabbri *et al.*, 2019). These findings collectively indicate that inflammation and alterations in immune cell phenotypes occur in the ovary during chemotherapy, contributing to decreased ovarian reserve.

### Oxidation–reduction imbalance

Oxidative stress, characterized by an overproduction of ROS and/or a deterioration in antioxidant defenses, directly impacts the intraovarian environment (Zhang *et al.*, 2015). ROS serve as double-edged swords within ovary, functioning as signaling molecules that promote follicle growth, ovulation, and corpus luteum formation when maintained in balance with antioxidants (Timoteo-Ferreira *et al.*, 2021). However, when this balance is disrupted, oxidative stress ensues, leading to follicular atresia and decreased oocyte quantity and quality (Timoteo-Ferreira *et al.*, 2021). Antioxidants present in the ovarian microenvironment, such as glutathione (GSH), glutathione peroxidase (GPx), catalase (CAT), and superoxide dismutase (SOD), play crucial roles in scavenging ROS to protect ovarian cells from oxidative stress damage.

CTX treatment had been observed to elevate levels of oxidoreductases, specifically malondialdehyde (MDA) and lactate dehydrogenase (LDH), while simultaneously reducing activity of the key antioxidant enzymatic activities of GPx, CAT, and SOD in mouse ovaries, thereby inducing DNA damage and apoptosis of follicles (Huang *et al.*, 2019, 2023; Ding *et al.*, 2020; Khanmohammadi *et al.*, 2021; Feng *et al.*, 2022; Zhao *et al.*, 2022). Similarly, the level of MDA was significantly increased in CTX-treated rat ovaries, while the activities of the antioxidant enzymes SOD, CAT, GPx, and GSH were significantly decreased (Abdel-Raheem *et al.*, 2015; Khedr, 2015; Saleh and Mansour, 2016; Yang *et al.*, 2017; Abdelzaher *et al.*, 2021; Hassan *et al.*, 2021; Talebi *et al.*, 2022; Zheng *et al.*, 2022). Furthermore, CTX-induced accumulation of oxidoreductase compromised the mitochondrial functions within mouse follicles, evident through diminished ATP and mtDNA production, alongside an increase in the rate of mitochondrial membrane potential reduction (Chen *et al.*, 2022). Oxidative stress markers, 8-hydroxy-2′-deoxyguanosine (8-OHdG), nitrotyrosine (NTY), and 4-hydroxynonenal (4-HNE), in mouse ovaries increased significantly after CTX treatment, potentially a result of inhibiting the NF-E2-related factor 2 (NRF2)/heme oxygenase-1 (HO-1) and SOD2 antioxidant pathways (Chen *et al.*, 2021). Intriguingly, administering CTX in multiple smaller doses appeared to enhance antioxidant enzymes activities and lowered ROS and MDA levels in mouse ovaries compared to a single, larger dose (Athira *et al.*, 2020). Likewise, the damage to ovary caused by a single high dose of CTX was more severe than that caused by multiple smaller doses, despite the total amount of CTX administered being greater in the latter case. Concomitantly, the number of healthy follicles of all categories (primordial, primary, preantral, and antral follicles) and proliferating granulosa cells were also higher following multiple smaller doses of CTX treatment. ROS levels increased in mouse ovaries under CTX/BUL chemotherapy, which led to abnormal follicular development and infertility (Chen *et al.*, 2021; Zhang *et al.*, 2021; Peng *et al.*, 2023). Additionally, CTX/BUL treatment hindered mouse oocyte mitochondrial biogenesis and diminished ovarian expression of 8-OHdG, MDA, and peroxisome proliferator-activated receptor gamma coactivator 1-alpha (Dai *et al.*, 2022). Together, these findings underscore the pivotal role of CTX-induced oxidative stress in driving ovarian dysfunction.

The side effects of CIS have also received considerable attention. The side effects of CIS are associated with an excessive production of free radicals and ROS, such as superoxide and H_2_O_2_. Following CIS treatment, a decrease in the expression of antioxidants (SOD, GSH, CAT, Cu/Zn-SOD) and an increase in oxidized MDA and total nitric oxide (NOx) levels were observed in rat ovaries, suggesting that CIS induces ovarian toxicity through increased oxidative stress (Li *et al.*, 2013; Meng *et al.*, 2015; Chinwe *et al.*, 2018; Soyman *et al.*, 2018; Ibrahim *et al.*, 2021; Al-Shahat *et al.*, 2022; Mentese *et al.*, 2022; Dinc *et al.*, 2023). Similarly, high 4-HNE, NTY, 8-OHdG, and MDA levels, and low CAT and GPx levels were observed in ovarian tissue of CIS-treated mice (Biyik *et al.*, 2021; Wang *et al.*, 2024). Mitochondria primarily contribute to ROS generation, and excessive oxidative stress can precipitate mitochondrial damage. In mice subjected to CIS treatment, the substantial ROS production within the ovarian microenvironment led to diminished numbers of active mitochondria and triggered follicular apoptosis (Chen *et al.*, 2015; Barberino *et al.*, 2017; Lins *et al.*, 2020; Gouveia *et al.*, 2021). Taken together, these findings underscore the association between CIS-induced ovarian toxicity and the destruction of follicles-mediated oxidative stress.

Some studies have indicated that ovarian toxicity of DOX is associated with ROS accumulation. DOX treatment resulted in a reduction in the mRNA expression of SOD and GSH while increasing the mRNA expression of oxidative stress-related genes, such as MDA and NRF2 in mouse ovaries (Niringiyumukiza *et al.*, 2019; Wang *et al.*, 2020). The stress induced by DOX prompted a protective response within mouse ovary, as shown by the upregulation of mRNA expression of HO-1 and CAT (Herrero *et al.*, 2023). Another study showed that DOX administration led to increased MDA and NOx levels, decreased SOD level, and induced oxidative DNA damage in rat ovaries (Morsi *et al.*, 2023). In conclusion, these studies indicate that DOX-induced ovarian damage is closely linked to disruptions in oxidative stress disturbance within the ovarian microenvironment.

### Ovarian stem cell exhaustion

The key significance of ovarian stem cells lies in their ability to sustain neo-oogenesis and replenish the primitive follicle pool within adult ovaries (Bukovsky and Caudle, 2012). The self-renewal and differentiation of stem cells are intricately governed by the homeostasis of their surrounding microenvironment or niche, especially the levels of oxidative stress and inflammation (Gattazzo *et al.*, 2014; Luo *et al.*, 2019). In the face of genotoxic stress induced by chemotherapy, ovarian stem cells are continuously compelled to proliferate and undergo accelerated depletion.

A recent study has discovered that the levels of the germline stem-specific markers mouse vasa homolog (MVH) and octamer-binding transcription factor 4 (OCT4) are significantly reduced in mouse ovaries following CTX treatment, indicating a depletion of ovarian stem cells (Jiang *et al.*, 2019). Subsequent research by the same group reported a near disappearance of ovarian germline stem cells in a CTX-induced POF mouse model, evidenced by diminished expression of MVH and OCT4 (Jiang *et al.*, 2019). Moreover, the activity of Hedgehog (Hh) signaling, which governs ovarian germline stem cell proliferation and stemness, was found to decrease in ovaries postchemotherapy. CTX/BUL chemotherapy induced apoptosis and dysfunction of mouse ovarian stem cells, potentially attributable to excessive oxidative stress in the ovarian microenvironment (Lai *et al.*, 2015; Sriraman *et al.*, 2015; Wu *et al.*, 2019). Interestingly, adriamycin, bleomycin, vinblastine, and dacarbazine (ABVD) treatment did not deplete human ovarian reserve and might even paradoxically increase the population of non-growing follicles. Post-ABVD tissue exhibited the presence of biovular and binucleate follicles, a feature more commonly associated with the prepubertal ovary (McLaughlin *et al.*, 2017). An alternative explanation may be that the ABVD combination, or specific components thereof, may activate germline stem cells to form oocytes or oocyte-like structures.

Despite its potential as a groundbreaking advancement in human reproductive science, the discovery of ovarian stem cells remains contentious. Nevertheless, it opens doors to innovative approaches in addressing ovarian dysfunction induced by chemotherapy. Although this area of research is still nascent, there remains ample opportunity to delve deeper into the biology of ovarian stem cells and explore their potential clinical application in fertility protection.

### Cell senescence

Cytotoxic drugs stimulate persistent DNA damage response signaling, which results in irreparable DNA damage and induces cell senescence (Spears *et al.*, 2019). As cells enter a senescent state, they secrete a range of cytokines, growth factors, inflammatory mediators, and proteinases, collectively referred to as the SASP. The abnormal accumulation of senescent cells within a tissue will cause cycle arrest, constrains the regenerative capacity of stem cells, and generates a proinflammatory milieu, ultimately driving senescence and dysfunction in normal cells and adjacent tissues (Calcinotto *et al.*, 2019).

Chemotherapy has been shown to trigger premature cellular senescence, exemplified by various studies. In a POF mouse model, CTX significantly inhibited proliferation and induced senescence in ovarian granulosa cells accompanied by heightened expression of p53, p66Shc, and p16, potentially through the activation of the long non-coding RNA-Meg3-p53-p66Shc pathway (Xiong *et al.*, 2017; Ai *et al.*, 2023; Xu *et al.*, 2023). Treatment with CTX/BUL led to an increase in senescent ovarian stromal cells in mice, concomitant with significant upregulation of cell cycle inhibition-related genes, such as p53, p21, and p27 (Dai *et al.*, 2022). Cell senescence had also been implicated in CIS-induced ovarian damage in mice, resulting in reduced oocyte quality and infertility (Marcozzi *et al.*, 2019; Du *et al.*, 2022). Furthermore, DOX treatment induced the accumulation of senescent cells in mouse ovaries, as evidenced by increased positive staining of β-gal and elevated expression of p16 and p21 in the ovarian stroma microenvironment, potentially contributing to ovarian dysfunction and infertility (Gao *et al.*, 2023).

Senescent cells within ovary have been found to secrete numerous proinflammatory factors, chemokines, and growth factors through autocrine or paracrine pathways (Anerillas *et al.*, 2020; Lopez-Otin *et al.*, 2023). The SASP creates specific microenvironments within mouse ovaries characterized by elevated levels of oxidative stress and inflammation (Hense *et al.*, 2022). Du *et al.* (2022) identified heightened SASP-related factors, particularly IL-6 and IL-1β, in CIS-treated mouse ovaries (Du *et al.*, 2022). Similarly, another study demonstrated increased expression of several common SASPs, such as IL-6, MCP-1, and TGF-β1, in mouse ovaries following DOX treatment (Gao *et al.*, 2023). Collectively, these findings underscore that senescent cells and SASPs contribute to the establishment of a chronic inflammatory state and ovarian damage during chemotherapy.

## Targeting the ovarian microenvironment to protect against chemotherapy-associated ovarian damage

**Figure 3.**
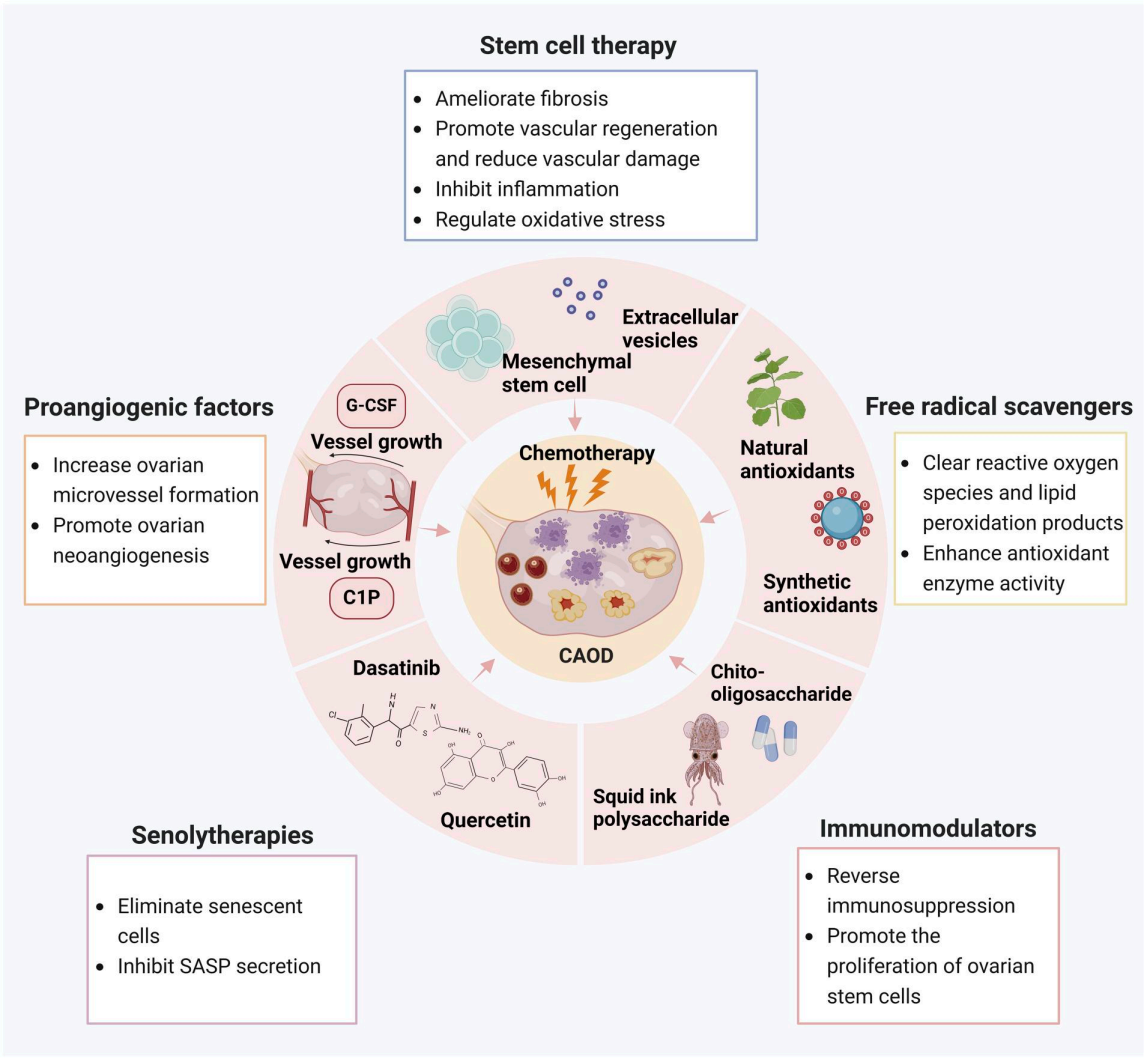
**Potential approaches targeting the ovarian microenvironment to protect against chemotherapy-associated ovarian damage.** CAOD, chemotherapy-associated ovarian damage; C1P, ceramide-1-phosphat; G-CSF, granulocyte/colony-stimulating factor; SASP, senescence-associated secretion phenotype. Created with BioRender.com, with permission.

Chemotherapy disrupts the delicate balance of the ovarian microenvironment, leading to poor oocyte developmental competence, follicular atresia, abnormal steroidogenesis, and infertility. Various strategies have been developed in mouse models to mitigate the ovarian damage caused by chemotherapy by targeting the ovarian microenvironment. These include stem cell therapy, free radical scavenging, immunomodulation, senolytherapies, and proangiogenic factors (Fig. 3). However, none of these approaches have been clinically tested for their efficacy in protecting against ovarian damage in humans undergoing chemotherapy.

### Stem cell therapy

Stem cells possess the remarkable ability to self-renew and differentiate into specific tissues according to their surrounding environment and signals (Na and Kim, 2020). Stem cells and their exosomes have demonstrated promising effects in improving various aspects of the ovarian microenvironment during chemotherapy. These effects include prevention of stromal fibrosis, preservation of blood vessel function, attenuation of inflammatory responses, and inhibition of oxidative stress (Table 2).

**Table 2.** Stem cell therapy to protect the ovarian microenvironment during chemotherapy.

Ovarian microenvironment	Categories	Drug	Model	Finding	Reference
Improved ovarian fibrosis	hUMSCs	CTX	Mouse	Decreased ovarian tissue fibrosis.	Zhou *et al.*, 2021
	hAECs	CTX	Mouse	Inhibited ovarian fibrosis and promoted ECM remodeling.	Huang *et al.*, 2023
	hUMSCs	CTX/BUL	Rat	Inhibited ovarian collagen deposition.	Chen *et al.*, 2023
	hUMSCs	CIS	Rat	Downregulated the expression of α-SMA, Collagen I, and III.	Cui *et al.*, 2020
	MenSCs	CTX	Rat	Reduced ovarian fibrosis by regulating SMAD2/4/6 pathway.	Yamchi *et al.*, 2021
	MenSCs	CIS	Mouse	Secreted FGF2 to ameliorate ovarian fibrosis.	Wang *et al.*, 2017
Promoted ovarian angiogenesis	BMSCs	CTX	Rabbit	Secreted VEGF to promote angiogenesis.	Abd-Allah *et al.*, 2013
	cMSCs	CTX	Mouse	Upregulated VEGF and IGF-1.	Eslami *et al.*, 2023
	hUMSCs	CTX	Mouse	Promoted ovarian angiogenesis by increasing the expression of VEGF-A.	Zhou *et al.*, 2021
	hAECs	CTX	Mouse	Promoted the expression of angiopoietin-like factor and CXCL16.	Huang *et al.*, 2023
	hUMSCs	CIS	Rat	Promoted the expression of VEGF, IGF-1, and FGF.	Qu *et al.*, 2022
	hUMSCs	CTX/BUL	Mouse	Promoted the expression of angiogenic cytokines VEGF, IGF, and angiogenin through activation of the PI3K/AKT pathway.	Yang *et al.*, 2019
	hUMSCs	CTX/BUL	Rat	Increased the expression of CD34, VEGF, and HGF to promote ovarian angiogenesis.	Chen *et al.*, 2023
	hAECs	CTX/BUL	Mouse	Secreted VEGF, TGF-β1, GDF9, and BMP15 to promote ovarian angiogenesis.	Yao *et al.*, 2016; Zhang *et al.*, 2017
	ES-MSCs	CTX/BUL	Mouse	Increased the secretion of VEGF, IGF-2, and HGF.	Bahrehbar *et al.*, 2020
Reduced ovarian inflammation	hAMSCs	CTX	Rat	Decreased the expression of IL-1β, IL-6, and TNF-α.	Ling *et al.*, 2017
	cMSCs	CTX	Mouse	Decreased TNF-α and IL-8 levels.	Eslami *et al.*, 2023
	hAECs	CTX	Mouse	Secreted 34 immune factors to regulate ovarian immune response.	Zhang *et al.*, 2017
	hUMSCs	CTX	Mouse	Decreased the expression of IL-6 and IL-1β, increased the expression of IL-10, TSG-6, and VEGF, and reduced the infiltration of neutrophils and macrophages.	Deng *et al.*, 2021
	SMSCs	CTX	Mouse	Reduced the activity of TNF-α, TGF-β, IL-8, IL-6, IL-1β, and IFN-γ.	Lai *et al.*, 2014
Restored ovarian oxidative stress balance	fMSCs	CTX	Mouse	Reduced the level of ROS.	Huang *et al.*, 2019
	hUMSCs	CTX	Mouse	Reduced ROS accumulation.	Ding *et al.*, 2020
	hAMSCs	CTX	Mouse	Inhibited the level of ROS.	Ding *et al.*, 2020
	hAECs	CTX	Mouse	Upregulated the level of antioxidant protein thioredoxin1/2, and reduced ROS.	Huang *et al.*, 2023

AKT, serine-threonine kinase; α-SMA, α-smooth muscle actin; BUL, busulphan; BMSCs, bone marrow mesenchymal stem cells; BMP15, bone morphogenetic protein 15; CTX, cyclophosphamide; CIS, cisplatin; CXCL16, CXC motif chemokine ligand 16; cMSCs, clonal mesenchymal stem cells; ECM, extracellular matrix; ES-MSCs, embryonic stem cell-derived mesenchymal stem cells; fMSCs, liver fetal mesenchymal stem cells; FGF, fibroblast growth factor; GDF9, growth-differentiation factor 9; hUMSCs, human umbilical cord mesenchymal stem cells; hAECs, human amniotic epithelial cells; hAMSCs, human amniotic mesenchymal stem cells; HGF, hepatocyte growth factor; IGF-1, insulin-like growth factor-1; IGF-2, insulin-like growth factor-2; IFN-γ, interferon gamma; MenSCs, menstrual blood-derived mesenchymal stem cells; PI3K, phosphoinositide 3-kinase; ROS, reactive oxygen species; SMAD, small mothers against decapentaplegic; SMSCs, skin-derived mesenchymal stem cells; TNF-α, tumor necrosis factor-alpha; TGF-β1, transforming growth factor-beta1; TSG-6, TNF-stimulated gene 6; VEGF, vascular endothelial growth factor.

Stem cells are capable of tissue regeneration and repair through the secretion of cytokines and extracellular vesicles. They play an important therapeutic role in combating fibrosis in various organs. In mouse models of CTX-induced POF, matrigel scaffolds laden with human umbilical cord mesenchymal stem cells (hUMSCs) effectively decreased the tissue fibrosis ratio by regulating the TGF-β1 pathway (Zhou *et al.*, 2021). Activated human amniotic epithelial cell (hAECs) transplantation produced MMP2 and MMP9 and played important roles in inhibiting fibrosis and promoting ECM remodeling in CTX-induced POI mouse ovaries (Huang *et al.*, 2023). Similarly, in a CTX/BUL-induced rat POF model, hepatocyte growth factor (HGF)-modified hUMSCs with overexpression of HGF exhibited superior inhibition of ovarian collagen deposition compared to hUMSCs-Null, attributable to the antifibrotic effect of HGF (Chen *et al.*, 2023). In a rat model of POI induced by CIS, the expression of the fibrosis markers α-SMA, Col-I, and Col-III was significantly inhibited following hUMSCs transplantation (Cui *et al.*, 2020). hUMSCs are shown to modulate the differentiation of ovarian stromal cells through the TGF-β1/SMAD3 signaling pathway, thus alleviating ovarian fibrosis. Additionally, menstrual blood-derived MSCs (MenSCs) demonstrated efficacy in reducing rat ovarian fibrosis induced by CTX by regulating the SMAD2/SMAD4/SMAD6 pathway (Yamchi *et al.*, 2021). In another study, MenSCs transplantation ameliorated mouse ovarian fibrosis induced by CIS through a paracrine mechanism, mediated by the secretion of FGF-2 (Wang *et al.*, 2017). These findings underscore the therapeutic potential of stem cells in mitigating ovarian fibrosis and preserving ovarian function in CAOD.

Stem cells exhibit remarkable regenerative potential by secreting various biological agents that promote angiogenesis. In a rabbit model of CTX-induced ovarian failure, bone marrow MSCs improved ovarian regeneration by secreting VEGF, thus promoting angiogenesis (Abd-Allah *et al.*, 2013). Similarly, clonal MSCs (cMSCs) and their secreted extracellular vesicles (EV20K and EV110) improved angiogenesis in mouse ovaries during CTX chemotherapy by upregulating VEGF and IGF-1 (Eslami *et al.*, 2023). Furthermore, hUMSCs combined with Matrigel promoted ovarian angiogenesis in a CTX-induced mouse POF model by upregulating VEGF-A (Zhou *et al.*, 2021). Additionally, hAECs, pre-stimulated by TNF-α and IFN-γ, significantly increased the production of proangiogenic factors (ANGPT-like factor and CXC motif chemokine ligand 16 (CXCL16)) in CTX-induced POI mouse ovaries (Huang *et al.*, 2023). In a CIS-induced POF rat model, hUMSC-derived exosomal microRNA (miR)-126-3p enhanced the expression of the angiogenesis-related factors VEGF, IGF-1, and FGF to improve ovarian function (Qu *et al.*, 2022). Similarly, transplantation of hUMSC-derived microvesicle (hUMSC-MV) during CTX/BUL chemotherapy promoted the expression of the angiogenic cytokines VEGF, IGF-1, and ANGPT in mouse ovaries through activation of the PI3K/AKT signaling pathway (Yang *et al.*, 2019). Notably, HGF-modified hUMSCs exhibited superior induction of CD34, VEGF, and HGF expression compared to the hUMSCs-Null group, indicating enhanced promotion of ovarian angiogenesis in CTX/BUL-induced POI rats (Chen *et al.*, 2023). Furthermore, hAECs secreted cytokines, such as VEGF, TGF-β1, growth-differentiation factor 9, and BMP15, to promote mouse ovarian angiogenesis after CTX/BUL-induced injury (Yao *et al.*, 2016; Zhang *et al.*, 2017). Embryonic stem cell-derived MSCs demonstrated restorative effects in CTX/BUL-induced POF mice through increased secretion of VEGF, IGF-2, and HGF from the ovaries (Bahrehbar *et al.*, 2020). These findings underscore the potential of various stem cell therapies to enhance ovarian angiogenesis and mitigate CAOD.

In addition to their progenitor characteristics, stem cells have unique immunomodulatory properties that provide new opportunities for the treatment of CAOD. Human amniotic MSCs (hAMSCs) decreased the expression of proinflammatory cytokines (IL-1β, IL-6, and TNF-α), thereby attenuating CTX-induced rat ovarian inflammation (Ling *et al.*, 2017). cMSCs and their secreted extracellular vesicles (EV20K and EV110) reversed the elevated levels of TNF-α and IL-8 in the ovaries of a CTX-induced mouse POF model (Eslami *et al.*, 2023). Furthermore, hAECs were found to secrete 34 immune factors that regulate the ovarian immune response and follicle development in CTX-induced POF mice (Zhang *et al.*, 2017). In mice treated with CTX, hUMSC transplantation resulted in the downregulation of proinflammatory factors IL-6 and IL-1β, upregulation of anti-inflammatory factors IL-10, TSG-6, and VEGF, and a reduction in the infiltration of neutrophils and macrophages into the ovary (Deng *et al.*, 2021). Furthermore, skin-derived MSC transplantation alleviated the activity of the inflammatory cytokines TNF-α, TGF-β, IL-8, IL-6, IL-1β, and IFN-γ, modulating the mouse ovarian inflammatory response and promoting a higher rate of oogenesis (Lai *et al.*, 2014). These findings underscore the immunomodulatory potential of various stem cell therapies in mitigating chemotherapy-induced ovarian inflammation and preserving ovarian function.

Indeed, stem cell therapy has demonstrated remarkable efficacy in the treatment of CAOD, with oxidative stress mitigation being one of key mechanism underlying their effectiveness. Liver fetal MSCs had been shown to prevent CTX-induced mouse follicle loss and restore sex hormone levels by reducing oxidative damage, enhancing oxidative protection, and restoring ovarian oxidative stress balance (Huang *et al.*, 2019). In a CTX-induced POI mouse model, exosomal miR-17-5P from hUMSCs restored ovarian function, and repressed ROS accumulation by downregulating the expression of Sirtuin 7 (SIRT7) (Ding *et al.*, 2020). Similarly, exosomal miR-320a from hAMSCs inhibited ROS level in CTX-induced POI mouse ovaries (Ding *et al.*, 2020). Additionally, activated hAECs transplantation significantly upregulated the expression of the antioxidant proteins thioredoxin1/2 and downregulated the expression of ROS in CTX-treated mouse ovaries (Huang *et al.*, 2023). These findings underscore the crucial role of stem cells in alleviating oxidative stress and restoring ovarian function in the context of CAOD.

Despite the extensively demonstration of efficacy in rodent models, there is a notable absence of clinical evidence supporting the use of stem cell transplantation as a safe clinical therapeutic option. While there are five registered clinical trials studies (NCT03166189, NCT02043743, NCT03816852, NCT03069209, NCT03877471) listed in the US National Institutes of Health clinical trial database (www.clinicaltrials.gov) investigating the use of stem cells to treat female infertility, all studies are still under investigation and have not progressed to phase III. Besides, the optimization of extraction methods and the administration routes for stem cell remains in the exploratory stage. The maintenance of MSCs for more than five subpassages is exceedingly difficult owing to their heterogeneity and culture inefficiency (Lee *et al.*, 2018). Additionally, safety assessments for stem cell therapy remain a primary concern. Under transplantation, certain stem cells may lose their characteristic features and could potentially undergo risky changes, such as gene mutation and modification, exposing patients to unknown harm (Marks *et al.*, 2017). Moreover, their limited availability, low survival rates of implanted cells, potential autoimmune responses, elusive signaling mechanism, and tumorigenicity associated with stem cell transplantation, as well as ethical obstacles, further complicate the therapeutic landscape. These challenges highlight the need for continued research and rigorous evaluation of stem cell therapy to ensure its safety and efficacy before clinical adoption.

### Free radical scavengers

Chemotherapy treatment induces an excessive accumulation of ROS within the ovarian microenvironment, leading to a significant increase in lipid peroxidation, depletion of intraovarian antioxidant, and significant DNA damage to cells (Sohal and Orr, 2012). Antioxidants, including natural antioxidants and synthetic antioxidants, play a crucial role as scavengers of these free radicals, helping to maintain the oxidant/antioxidant balance. Predominantly, natural antioxidants such as polyphenols (phenolic acids, flavonoids, and lignans) and carotenoids are found in a variety of foods and medicinal plants (Xu *et al.*, 2017), such as epigallocatechin gallate, theaflavins, *Pleurotus columbines*, *Lycium barbarum* polysaccharide, sesamol, crocin, resveratrol, pyrroloquinoline-quinine, chrysin, rutin, hesperidin, *Nigella sativa*, silibinin, lycopene, and coenzyme Q10. In addition to these natural substances, a variety of synthetic compounds known for their antioxidant capabilities have been explored for their potential to counteract ROS, such as irbesartan, fenofibrate, mirtazapine, *N*-acetyl-l-cysteine, erythropoietin, mesna, ebselen, hydrogen-rich saline, melatonin (*n*-acetyl-5-methoxytryptamine), and several formulations of traditional Chinese medicine (erxian decoction and modified Dihuang decoction).

#### Natural antioxidants

Natural antioxidants play a pivotal role in safeguarding against ovarian damage during CTX chemotherapy by effectively scavenging excess ROS through various pathways. Epigallocatechin gallate and theaflavins, prominent polyphenols derived from green or black tea, increased the expression of antioxidant enzymes by activating NRF2/HO-1 pathways, and alleviated CTX-induced ovarian oxidative stress and fibrosis in mice (Chen *et al.*, 2021; Barberino *et al.*, 2022). Likewise, *Pleurotus columbinus* extracts, rich in phenolic and flavonoid compounds, exhibited protective effects against CTX-induced rat ovarian damage by reducing lipid peroxidation levels and enhancing the antioxidant activity (Hassan *et al.*, 2021). *Lycium barbarum* polysaccharide, a key component extracted from the Lycium barbarum plant, showcases antioxidant properties attributed to its constituents such as carotenoids, flavonoids, ascorbic acid and its derivatives, and polyphenols (Tian *et al.*, 2019). *Lycium barbarum* polysaccharide reduced oxidative stress, enhanced the activity of antioxidant enzymes, and reduced the level of oxidative products to protect rat ovarian function during CTX chemotherapy (Yang *et al.*, 2017). Similarly, in a rat POF model induced by CTX, the antioxidative enzymatic activity of SOD was increased and the MDA level was decreased after coadministration of sesamol or crocin with CTX (Khanmohammadi *et al.*, 2021; Talebi *et al.*, 2022). In another study, a low concentration of resveratrol or pyrroloquinoline-quinine attenuated the oxidative stress level in CTX/BUL-treated mouse ovaries, fostering a conducive microenvironment for oogonial stem cells (Wu *et al.*, 2019; Dai *et al.*, 2022). Notably, coenzyme Q10 administration during CTX treatment reduced ROS levels in mouse ovaries, correlating with increased oocyte quantity and quality (Delkhosh *et al.*, 2019). Together, these findings suggest that natural antioxidants may be promising drugs for protecting ovarian function, with potential clinical applications during CTX chemotherapy.

Five different natural antioxidants, namely chrysin, rutin, resveratrol, *Nigella sativa*, and hesperidin, have been tested for their protective effects on the ovaries of mouse and rat treated with CIS (Khedr, 2015; Chinwe *et al.*, 2018; Lins *et al.*, 2020; Ibrahim *et al.*, 2021; Mentese *et al.*, 2022; Cetinkaya *et al.*, 2023). Chrysin pretreatment decreased MDA, total oxidant status (TOS), and oxidative stress index and increased total antioxidant status (TAS) in ovaries during chemotherapy. The antioxidative enzymatic activity (MPO, SOD, and GPx) and GSH were increased after coadministration of flavonoid rutin, hesperidin resveratrol, or *Nigella sativa* with CIS. These results indicate that the scavenging of ROS in the ovary is important to protect the ovarian reserve during CIS chemotherapy.

Antioxidants also have a protective effect on ovarian damage induced by in other chemotherapy agents. 5-Fluorouracil is an antimetabolite drug and ovarian toxicity is one of the most important side effects. Silibinin, a natural flavonolignan, prevented 5-fluorouracil-induced oxidative damage in rat ovaries by decreasing TOS and increasing TAS (Ayazoglu Demir *et al.*, 2023). Lycopene, a type of carotenoid, increased the expression of GSH and decreased the expression of MDA in methotrexate-treated rat ovaries (Turkler *et al.*, 2020).

#### Synthetic antioxidants

Four synthetic antioxidants have been tested as ovarian protectants against CTX-induced damage. Irbesartan is a synthetic nonpeptide antagonist of angiotensin II. In addition to its effect of lowering blood pressure, irbesartan acts as a free radical scavenger (Wang *et al.*, 2013). Irbesartan pretreatment significantly prevented CTX-induced rat ovarian dysfunction by reducing oxidative stress (Abdel-Raheem *et al.*, 2015). Fenofibrate, a PPARα agonist, is reported to prevent rat ovarian damage induced by CTX through antioxidant actions (Abdelzaher *et al.*, 2021). Mirtazapine, an antidepressant drug, reversed the ratio of oxidase to antioxidant enzymes in rat ovary and protects fertility against CTX- and CIS-induced toxicity (Altuner *et al.*, 2013; Khedr, 2015). The *N*-acetyl-l-cysteine is a potent scavenger of free radicals, and it had been suggested to have beneficial effects in inhibiting ROS production and restoring GSH to normal level in human and rat ovaries exposed to chemotherapy, resulting in an improvement of follicle viability and pregnancy rate (Helal, 2016; Li *et al.*, 2019). The traditional Chinese medicine, modified Dihuang decoction, upregulated the levels of SOD and GPx in CTX/BUL-induced DOR mouse ovaries (Zhang *et al.*, 2021).

Synthetic antioxidants can also provide protection against CIS-induced oxidative stress along with underlying disorders in the ovary. In addition to promoting erythropoiesis, erythropoietin has antiapoptotic, antioxidant, anti-inflammatory, and angiogenic effects (Hardee *et al.*, 2006). Erythropoietin improved ovarian function by reducing CIS-induced oxidative stress levels in rat ovaries (Dayangan Sayan *et al.*, 2018). Mesna, a Food and Drug Administration (FDA)-approved antioxidant, prevents the toxic side effects of chemotherapy agents by removing ROS and upregulating antioxidant enzymes (Li *et al.*, 2013; Munoz-Osores *et al.*, 2022). Ebselen could improve CIS-induced rat ovarian damage by increasing SOD and GSH levels and reducing MDA and NOx levels (Soyman *et al.*, 2018). Hydrogen exerts a therapeutic antioxidant effect by selectively alleviating oxidation products and improving the activity of antioxidants (Xie *et al.*, 2010; Qu *et al.*, 2012). Hydrogen-rich saline treatment reversed the effect of CIS on rat ovarian MDA, SOD, and CAT (Meng *et al.*, 2015). Melatonin is mainly secreted by the vertebrate pineal gland and has antioxidation characteristics (Reiter *et al.*, 2013, 2014). The ovarian protective effect of exogenous melatonin in CIS-treated mice could be attributed to its antioxidant activity, manifested as scavenging ROS and stimulating antioxidant activity (Barberino *et al.*, 2017; Huang *et al.*, 2021; Al-Shahat *et al.*, 2022). Erxian decoction of a Chinese herbal formula reduced the amount of MDA and raised the activity of SOD in a CIS-induced rat POF model (Li *et al.*, 2007; Liu *et al.*, 2023). However, the antioxidant mechanisms of traditional Chinese medicines are complex and need further study.

Cancer is also characterized by increased oxidative stress, which can initiate tumor development and contribute to tumor progression by directly oxidizing macromolecules or through oxidative stress-induced aberrant redox signaling (Canli *et al.*, 2017). It is an attractive idea to use antioxidants for cancer treatment and some antioxidants, namely resveratrol and ebselen, have been explored in clinical research (such as breast cancer, neuroendocrine tumor, multiple myeloma, head and neck cancer, and lung cancer) (Luo *et al.*, 2022). However, the major challenge with natural antioxidants is their unstable chemical structure, low oral bioavailability, limited aqueous solubility, low targeted efficacy, and potential hepatotoxicity and nephrotoxicity at high doses; the latter is being targeted for improvement by encapsulating natural antioxidants in nano-sized vehicles for further delivery. Nevertheless, there are still few studies on the safety of natural antioxidant-delivery nanosystems. Alternatively, multiple antioxidants are undergoing pre-clinical study, and high-quality clinical trials of antioxidants in CAOD are lacking. Moreover, safe dosage levels of antioxidants in the human ovary remain undefined. Besides, the molecular mechanism underlying the action of antioxidants against CAOD is not fully elucidated owing to the diverse structures and functionalities of these compounds. Therefore, future research should aim to determine whether antioxidants interfere with the effectiveness of chemotherapy against tumor cell growth. Large-scale, multi-center clinical trials are urgently needed to confirm the safety and antioxidant effect of antioxidants on human ovaries, providing crucial insights for the future perspectives in CAOD management.

### Immunomodulators

Immunomodulators are drugs that regulate immune function, primarily by stimulating immune cell activity or modulating the production of inflammatory factors in a nonspecific manner. They have the potential to improve the ovarian environment disrupted by chemotherapy, thus promoting follicular growth and recovery of ovarian function.

Chito-oligosaccharide (COS), derived from shrimp and crustacean through deacetylation, acts as a natural immune enhancer (Xia *et al.*, 2019). COS induces cytokine secretion by inducing the accumulation and activation of macrophages and polymorphonuclear cells, thus stimulating the immune system (Mudgal *et al.*, 2019). In a recent study, COS administration reversed the immunosuppression of the mouse ovarian microenvironment caused by CTX/BUL chemotherapy, preventing decreased levels of IL-2 and TNF-α and increased levels of IL-4 (Huang *et al.*, 2021; Li *et al.*, 2023). Furthermore, COS promoted the proliferation of mouse ovarian stem cells by regulating the secretion of the immune factors IL-2 and TNF-α (Huang *et al.*, 2021; Zheng *et al.*, 2023). Squid ink polysaccharide (SIP), a glycosaminoglycan isolated from *Sepia esculenta* ink, exhibits immunomodulatory properties by enhancing immune function (Zuo *et al.*, 2014). A study conducted in CTX-treated mice proposed that SIP ameliorated ovarian immunosuppression and increased IL-2 and TNF-α expression (Liu *et al.*, 2019).

Immunomodulatory therapeutics, aimed at activating the immune system for tumor suppression and restoring normal immune responses, hold potential for enhancing cancer therapy (Khalil *et al.*, 2016). Several reports have demonstrated that COS induces the death of cancer cells via repressing tumor growth, triggering the apoptosis signaling pathway, and regulating immunity; tumors responding to COS include lung cancer (Ngo *et al.*, 2019), liver cancer (Jing *et al.*, 2019), renal carcinoma (Zhai *et al.*, 2019), colorectal cancer (Han *et al.*, 2016), and osteosarcoma (Pan *et al.*, 2021). Additionally, SIP and its derivative exert their antitumor effects in various cancers, such as liver cancer (Tian *et al.*, 2023), ovarian carcinoma (Zong *et al.*, 2015), and melanoma (Zong *et al.*, 2013), which may be associated with its immunostimulating and proapoptotic activity. However, COS and SIP are relatively recent discoveries and require further investigation in order to elucidate the precise molecular mechanisms involved in their anticancer and ovarian protection effects. Clinical experiments are needed to evaluate the safety and efficacy of these immunomodulators in protecting the human ovary from chemotherapy-induced damage. In addition, comprehensive studies should evaluate whether immunomodulators have an adverse effect on the chemotherapy drug to be used as an adjuvant.

### Senolytherapies

Chemotherapy is acknowledged as a common stressor known to induce cellular senescence, with senescent cell-secreted SASPs and exacerbating inflammation in the ovarian microenvironment, potentially driving the progression of CAOD (Gao *et al.*, 2023). Senolytics have emerged as promising agents for selectively targeting senescent cells through the activation of ‘suicide’ genes and the modulation of various cellular pathways (Baker *et al.*, 2011). Recent studies display potential for enhancing female fertility during chemotherapy.

Dasatinib (D) and quercetin (Q), both natural flavonoids and the most typical senolytic agents, bind to B-cell lymphoma-2 (BCL-2) and regulate transcription factors, cyclins, proapoptotic and antiapoptotic proteins, and growth factors (Kirkland and Tchkonia, 2020). In clinical trials, D + Q combination therapy has demonstrated efficacy in reducing senescent cell burden and improving physical performance (Hickson *et al.*, 2019). In CTX-induced POI model mice, the increased expression of the cellular senescence markers p16, p21, p53, and γ-H2AX in granulosa cells was reversed by cotreatment with D + Q (Xu *et al.*, 2023). Similarly, fisetin exhibits senolytic properties and shows promise in mitigating age-related pathology and extending lifespan (Yousefzadeh *et al.*, 2018). Our group demonstrated that short-term intervention with D + Q or fisetin significantly reduced senescent cell accumulation in mouse ovary during DOX treatment, yet failed to reverse DOX-induced follicle loss and ovarian stromal fibrosis caused by DOX (Gao *et al.*, 2023). However, in a separate study, D + Q effectively reversed ovarian fibrosis in CIS-exposed mice by removing senescent cells (Du *et al.*, 2022). The varying degrees of ovarian cell senescence induced by different chemotherapy drugs highlights the complexity of utilizing senolytics for CAOD protection.

Some senotherapeutic drugs, such as quercetin, dasatinib, and fisetin, offer promising anticancer effects with minimal adverse effects and high efficacy (Mamun *et al.*, 2022). Therefore, senotherapeutics not only hold potential for preserving ovarian function but also exert antitumor effects during chemotherapy. However, identifying the mechanistic actions of senotherapeutics, especially *in vivo*, remains challenging and is influenced by factors such as senescent cell type and drug concentration. Formal assessment of their efficacy and adverse effects in human clinical trials is imperative considering the inherent differences between animal models and humans. Additionally, preserving beneficial senescent cell populations is crucial, as they play vital roles in tissue renewal, wound healing, and cancer prevention. Further research is warranted to explore the impact of senotherapeutics on antitumor activity while safeguarding the ovarian reserve from chemotherapy-induced gonadotoxicity.

### Proangiogenic factors

Chemotherapy drugs inhibit ovarian angiogenesis or destroy the structure and function of blood vessels, resulting in ovarian dysfunction. Proangiogenic factors are vital molecules that enhance tissue vascularization within the perivascular and vascular microenvironment (Hamid and Mirshafiey, 2016).

Granulocyte CSF (G-CSF) is a glycoprotein that induces VEGF expression and secretion, promoting angiogenesis through regulating the PI3K/AKT pathway. In CTX/BUL-induced POI mouse ovaries, G-CSF treatment improved follicular development and fertility by promoting ovarian microvessel formation (Skaznik-Wikiel *et al.*, 2013; Buigues *et al.*, 2021). Similarly, treatment with G-CSF increased ovarian neoangiogenesis, leading to a significant increase in follicle number and serum anti-Müllerian hormone level in rats treated with CIS (Akdemir *et al.*, 2014). Besides, G-CSF-mobilized peripheral blood mononuclear cells combined with platelet-rich plasma increased ovarian angiogenesis and the expression of VEGF and CD34 in CTX-induced POI rats (Huang *et al.*, 2019). Ceramide-1-phosphate (C1P), a potent sphingolipid released by damaged tissue cells, modulates vascular development and apoptosis in ovaries affected by chemotherapy (Kim *et al.*, 2013). In a CTX-induced ovarian damage mouse model, C1P restored damaged stromal vascular structures and the continuity of the endothelial layer, thereby promoting ovarian vascular stability (Pascuali *et al.*, 2018). Clinical studies assessing the effects of G-CSF and C1P treatment on CAOD are warranted, alongside investigations to reliably evaluate their ovarian-protective effects and potential side effects.

While proangiogenic factors have a protective role against CAOD, their impacts on tumors need to be considered. Promotion of angiogenesis may inadvertently stimulate tumor growth and metastasis, influencing the efficacy of chemotherapy. Therefore, tumor-bearing models should be utilized to explore the protective effect of proangiogenic factors on CAOD and their effects on tumors. Furthermore, elucidating the precise molecular mechanisms involved in the action of proangiogenic factors through well-designed experiments is crucial for understanding their anticancer and CAOD protective effects.

## Conclusion

The risk of CAOD represents a critical concern regarding both short- and long-term adverse effects of anticancer treatments in premenopausal women. According to current guidelines, it is imperative that all premenopausal women undergoing cancer therapy engage in comprehensive oncofertility counseling. This is essential to preserve normal endocrine functionality and to ensure the feasibility of fulfilling their family aspirations. The ovarian microenvironment, serving as the foundational milieu for follicle development, experiences profound alterations in response to chemotherapy. A substantial body of evidence, including clinical studies, human ovarian xenograft research, and mouse model investigations, suggests that certain chemotherapeutic agents may induce changes, such as ECM accumulation and fibrosis, disturbances in ovarian angiogenesis, disruptions in the immune microenvironment, imbalances in oxidative stress homeostasis, ovarian stem cell depletion, and cellular senescence, thereby adversely affecting the quantity and quality of ovarian follicles.

A notable gap in awareness exists regarding the changes to the ovarian microenvironment induced by chemotherapy, including alterations in ovarian lymphatic vessels, nerves, metabolic products, phenotypic and functional shifts in specific stromal cell populations, and their interactions with folliculogenesis, follicle positioning, and hormone synthesis. Advanced techniques, such as single-cell and spatial transcriptomics, could help to elucidate the shifts in cellular composition and the pathways implicated in the impact of chemotherapy. Given the limitations associated with animal models, further research is crucial to delineate the effects of chemotherapeutic agents on the human ovarian microenvironment, particularly to distinguish between the mechanisms involved in the immediate and delayed phases of chemotherapy-induced ovarian imbalance.

While current protective agents primarily focus on averting ovarian follicle loss, it is vital to acknowledge the role of the stromal environment in follicle health, beyond the direct follicular impact. A strategy of targeting the ovarian microenvironment for CAOD treatment is emerging, albeit in its early stages. MSC transplantation, for example, demonstrates promise in mitigating CAOD through enhancements in the ovarian microenvironment, such as reducing fibrosis, fostering angiogenesis, modulating immunity, and alleviating oxidative stress damage, with significant efficacy in animal models yet pending clinical testing/application. Furthermore, the exploration of antioxidants, immunomodulators, senolytics, and proangiogenic factors as novel CAOD protectants necessitates more research to elucidate the exact molecular mechanisms for their combined anticancer and ovarian protective effects. Like stem cell therapies, these approaches currently lack substantial clinical evidence to be considered safe therapeutic options.

Compiling this review has highlighted the challenges, complexities, and uncertainties of transitioning protectants from laboratory settings to clinical application, particularly emphasizing the paramount concern of safety for cancer patients undergoing treatment for malignant conditions. Protectants must not only prevent CAOD but also demonstrate no adverse interference with the efficacy of tumor chemotherapy. Moreover, assessing the clinical efficacy of these protective measures remains challenging, as potential protectants might reduce rather than completely prevent ovarian damage, leaving the impact on future fertility uncertain.

Despite these translational challenges, this review underscores the significance of addressing changes in the ovarian microenvironment during chemotherapy and the development of novel therapeutics targeting this microenvironment to enhance ovarian function. It is our hope that this review will foster professional discourse and inspire future research directions in this field.

## Data Availability

No new data were generated or analyzed in support of this research.
